# Changes in androgen profile over the menstrual cycle and hormonal contraceptive phases in physically active females

**DOI:** 10.1186/s12905-025-04253-6

**Published:** 2026-01-27

**Authors:** Vera M. Salmi, Ritva S. Mikkonen, Ida E. Löfberg, Kelly L. McNulty, Kirsty M. Hicks, Anthony C. Hackney, Johanna K. Ihalainen

**Affiliations:** 1 Faculty of Sport and Health Sciences, University of Jyväskylä, Jyväskylä, Finland; 2 Department of Sport, Exercise and Rehabilitation, Faculty of Health and Life Sciences, Northumbria University, Newcastle-upon-Tyne, UK; 3 Performance, Medical and Innovation Department, Washington Spirit Soccer Club, Washington, DC USA; 4 Department of Exercise & Sport Science – Department of Nutrition, University of North Carolina, Chapel Hill, NC USA; 5 Finnish Institute of High Performance Sport KIHU, Jyväskylä, Finland

**Keywords:** Menstrual cycle, Hormonal contraceptives, Female physiology, Sex hormones, Androgens

## Abstract

**Background:**

Concentrations of female sex hormones fluctuate during the menstrual cycle (MC), while hormonal contraceptives (HC) generally suppress hypothalamic-pituitary-ovarian axis function resulting in lower concentrations of endogenous estradiol (E2) and progesterone (P4). Little attention has been paid to changes in androgen concentrations during MC and HC phases. Therefore, the aim of this study was to investigate changes in androgen concentrations over the MC and HC phases.

**Methods:**

The study was a cross-sectional analysis across MC and HC phases using four time points. E2, P4, follicle-stimulating hormone (FSH), luteinizing hormone (LH), total testosterone (tT), free testosterone (fT), sex hormone-binding globulin (SHBG), dehydroepiandrosterone (DHEA) and its sulfate (DHEA-S) were analyzed from the serum of healthy, physically active, naturally menstruating (NM = 36), hormonal intrauterine device using (IUD = 12) and combined HCs using (CHC = 25) females.

**Results:**

In NM, concentrations of tT (β = 0.16, *p* = 0.050), fT (β = 1.85, *p* < 0.001) and DHEA (β = 5.29, *p* = 0.045) were significantly higher at ovulatory phase and concentrations of DHEA-S at the mid-follicular phase (β = 0.32, *p* = 0.012) compared to bleeding. In IUD, tT concentrations fluctuated significantly being highest at mid-cycle (β = 0.34, *p* = 0.001) compared to the sample defined as measurement 1 (based on bleeding and/or hormonal concentrations). In CHC, concentrations of E2, P4, tT, fT, DHEA, and DHEA-S remained unchanged between the HC phases.

**Conclusions:**

Since the endogenous hormonal milieu, including androgens, may affect female physiology, considering the fluctuation in androgen levels over the MC and HC phases may be of importance in physiological research.

**Supplementary Information:**

The online version contains supplementary material available at 10.1186/s12905-025-04253-6.

## Background

The major estrogen and progestogen secreted by the ovaries are estradiol (E2) and progesterone (P4) [1–3]. In addition to these sex steroids, the ovaries secrete androgens, such as testosterone (T) and precursors to T such as androstenedione (A4) and dehydroepiandrosterone (DHEA) [1–3], which have little androgenic activity, but can be peripherally converted to more potent androgens [4]. DHEA is predominantly derived from the adrenal cortex whereas A4 and T are derived from both the ovaries and the adrenal cortex [5]. A portion of T is converted to E2 through the action of an aromatase enzyme while most of the T left in the bloodstream is bound by sex hormone-binding globulin (SHBG), a binding protein for both androgens and estrogens that prolongs sex hormone circulating life [6, 7]. Ultimately, the free hormone fraction (fT) of total T (tT) in circulation is only 0.3–2% in females [8, 9].

Although androgens act as precursors for the biosynthesis of estrogens like E2, circulating androgens also have direct effects on various physiological systems outside of the reproductive system [10] including the cardiovascular and musculoskeletal systems [11]. These effects may be influenced by changes in female sex steroid concentrations [12], which, in turn, may influence e.g., metabolic substrate utilization and perceived exertion [13] as well as recovery from exercise [14]. Meta-analyses have shown that both strength and endurance exercise performance might be on average slightly inferior when both E2 and P4 are at their lowest during the early follicular phase (FP) of the menstrual cycle (MC) compared to other phases [15]. Likewise, performance appears to be slightly inferior in combined oral contraceptive pill (OC) users compared with eumenorrheic women, which may also be attributed to downregulated concentrations of endogenous E2 and P4 [16]. Since hormonal profiles of E2 and P4 during the early FP and combined OC use are comparable [17], it has been suggested that exercise performance might be mediated by concentrations of endogenous female sex hormones [15]. Regrettably, research thus far has not included the possible influence of changes in the androgen profile on these interactions.

A eumenorrheic MC is often divided into two main phases, separated by ovulation: (1) the FP; and (2) the luteal phase (LP). Concentrations of P4 > 16 nmol·L^−1^ and a positive luteinizing hormone (LH) surge test indicate eumenorrhea [18]. In previous research, T levels have been reported to be highest around the LH peak associated with ovulation [19–22]. Hormonal contraceptives (HC) are exogenously administered steroid hormones that act by means of influencing endogenous hormones, thus they can be used for contraception as well as the manipulation of menstruation [23] and/or alleviating the symptoms of dysmenorrhea [24, 25] and menorrhagia [26]. There are two main types of HCs; progestin-only HCs and combined HCs that contain an estrogen and progestin component [27]. Progestin-only HCs have several delivery methods, including pill, implant, and injection, which have a systemic effect preventing ovulation. In addition, progestin-releasing intrauterine devices (IUD) are available. These primarily act locally, affecting the milieu in the uterus, where low concentrations of levonorgestrel (LNG) are absorbed into systemic circulation and are insufficient to invariably affect ovulation [28]. The hormonal profile of a female using progestin-releasing IUD can differ from a normal ovulatory cycle to an anovulatory cycle, the latter of which is more frequently observed in females with higher serum levels of IUD-released progestin [28]. Combined HCs also include different delivery methods including the pill, vaginal ring, and transdermal patch [18]. These combined methods are typically administered for 21 to 24 days followed by a 4 to 7-day exogenous hormone-free phase. The estrogenic component typically used in combined HCs is ethinyl estradiol coupled with one of the many progestins available. In addition, there are few E2-containing preparations available which contain estradiol valerate or estradiol hemihydrate [29]. The hormonal profile of females using combined OCs is characterized by a daily surge of low-dose exogenous estrogen and progestin during the intake of the hormone-containing pills (active phase), accompanied by an increase in endogenous E2 during the exogenous hormone or pill-free phase (inactive phase) [16]. Combined OCs prevent ovulation and the cyclical variation in E2 and P4 by negative feedback suppressing the hypothalamic-pituitary-ovarian (HPO) axis function [30, 31].

The aim of this study was to investigate the changes in endogenous androgen and SHBG concentrations across the MC in physically active naturally menstruating females (NM) and across HC use in physically active females using hormonal intrauterine device (progestin-only HCs) (IUD) or combined hormonal contraceptives (CHC) at four time points. In addition, we compared hormone concentrations between these groups. To our knowledge, there are no studies that have investigated the effects of LNG-releasing IUDs on changes in androgen concentrations over the HC phases. Furthermore, we investigated if there were associations between E2 and P4 fluctuations and measured changes in androgen levels over the MC and HC phases. The hypothesis for this study was that androgen levels would be elevated near ovulation in NM and IUD. We hypothesized that in CHC, the androgen levels would be downregulated similar to E2 and P4 during the HC phases.

## Methods

### Participants

Participants were recruited by advertisements via the University of Jyväskylä’s website and social media channels as well as the local newspaper. Inclusion criteria: healthy, physically active (recreationally active or trained, tier 1–2) [32], 18–40 years old, BMI 18–25 kg∙m^−2^ females, who (1) had a regular MC and had not used HCs at least 3 months prior to the recruitment or females, who (2) were using either combined HCs or hormonal IUDs at least 3 months prior to the recruitment. Exclusion criteria: chronic diseases, endocrine disorders, medication (other than HCs in IUD and CHC), pregnancy, lactation, amenorrhea, known polycystic ovary syndrome or any other disease or conditions affecting ovarian function. The participants enrolled in the study completed a health questionnaire and received detailed information about the study design, measurements, procedures, and possible risks. The data presented is part of the *Endogenous and Exogenous Hormones and Performance in Women (MEndEx)* study [33–35] and *Recovery in Women Discordant for Hormonal Contraceptive Use (ReWo)* study [36]. This research was completed in accordance with relevant guidelines and regulations: the Ethical Committee of the University of Jyväskylä approved both studies (October 22, 2018, and August 30, 2019, respectively), written informed consent was obtained from each study participant before participation, and the study was conducted in accordance with the Declaration of Helsinki.

Participants made up three groups: (1) naturally menstruating (NM, *n* = 36); (2) hormonal intrauterine device (progestin-only hormonal contraceptive) using (IUD, *n* = 12); and (3) combined hormonal contraceptive using (CHC, *n* = 25) females. Participant characteristics are presented in Table 1. Participants in NM reported a natural MC based on bleeding for the previous 6 months and had not used a HC for at least one year. Participants in IUD and CHC had used HC for at least one year and they reported regular menstrual cycles before starting HC and had not experienced missed periods (amenorrhea) for reasons other than pregnancy. The participants in IUD used LNG-releasing hormonal IUDs and the participants in CHC used either third or fourth generation progestin or cyproterone acetate containing combined monophasic OCs, or contraceptive rings (Table 2). Active pills used by participants in CHC contained differing amounts of estrogenic (0.02–0.03 mg ethinyl estradiol or 1.5 mg bioidentical estradiol hemihydrate) and progestogenic (0.075–3.0 mg) compounds. The timing of these doses across the HC phases varied. The active phase ranged from 21 to 24 days followed by 4–7 hormone-free days (inactive phase).

**Table 1:** Participant characteristics in all three groups

Group	NM	IUD	CHC
Age (years)	25.8 ± 4.3	28.5 ± 6.4	24.5 ± 3.0
Height (m)	1.67 ± 0.06	1.69 ± 0.05	1.69 ± 0.05
Body mass (kg)	64.6 ± 7.1	62.6 ± 8.8	64.4 ± 5.6
BMI (kg∙m^−2^)	23.3 ± 2.5	21.9 ± 3.0	22.5 ± 2.0
V̇O_2peak_ (ml∙kg^−1^∙min^−1^)	45.1 ± 5.3	45.8 ± 5.8	43.9 ± 4.7
Length of menstrual cycle (days)	28 ± 3		

The incremental treadmill running test was performed using a standard incremental protocol to assess and describe the physical performance level of the participants [33, 36]

Values are means ± SD

*NM* Naturally menstruating females, *IUD* Hormonal intrauterine device using females, *CHC* Combined hormonal contraceptives using females, *BMI* Body mass index, *V̇O*_*2peak*_ Peak oxygen uptake

**Table 2:** Hormone components and brand names of hormonal contraceptives

	Pills/rings/IUDs included	Content (mg/mg)	Brand names (active phase + inactive phase)
IUD: Hormonal IUD			
	Levonorgestrel 13.5 mg (*n* = 2)		Jaydess
	Levonorgestrel 19.5 mg (*n* = 4)		Kyleena
	Levonorgestrel 52 mg (*n* = 6)		Mirena
*CHC: Third generation pills*			
	Ethinyl estradiol coupled with gestodene (*n* = 1)	0.02/0.075	Meliane (21 + 7)
	Ethinyl estradiol coupled with desogestrel (*n* = 2)	0.02/0.15	Mercilon and Lumivela (21 + 7)
*CHC: Fourth generation pills*			
	Ethinyl estradiol coupled with drospirenone (*n* = 11)	0.02/3	Yaz, Stefaminelle (24 + 4), Yasminelle, and Tasminetta (21 + 7)
		0.03/3	Yasmin (21 + 7)
	Ethinyl estradiol coupled with dienogest (*n* = 1)	0.03/2	Dienorette (21 + 7)
	Estradiol hemihydrate coupled with nomegestrol acetate (*n* = 3)	2.5/2.5	Zoely (24 + 4)
*CHC: Other pills*			
	Ethinyl estradiol coupled with cyproterone acetate (*n* = 2)	0.035/2	Vreya (21 + 7)
*CHC: Contraceptive rings*			
	Ethinyl estradiol coupled with etonogestrel (*n* = 4)	0.015/0.120	Nuvaring, Ornibel and Vagiprev (21 + 7)

Information of one participant from CHC was not reported

*IUD* Hormonal intrauterine device using females, *CHC* Combined hormonal contraceptive using females, *IUD* Intrauterine device

### Design

**Fig. 1:**
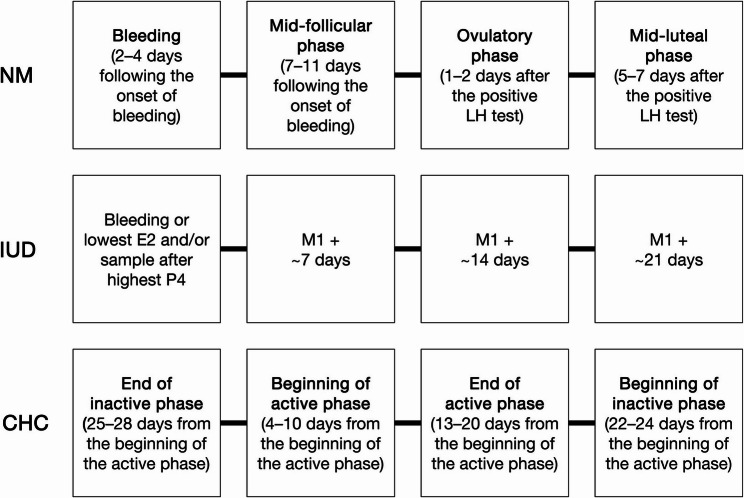
Overview of the timing of blood samples. NM, naturally menstruating females; IUD, hormonal intrauterine device using females; CHC, combined hormonal contraceptive using females

The study was a cross-sectional analysis across one MC in the NM and for approximately 4 weeks in IUD and CHC. Participants filled-in MC/HC diaries, which included information about bleeding days. Four testing sessions were completed over an individual MC or HC. The measurement time points for IUD and CHC are hereafter referred to as HC phases, even though active and inactive phases in CHC are “artificial” compared to the phases of the physiological MC [16]. The phase of the MC or HC use in which testing commenced was randomized. Time points for measurements in NM were at bleeding (M1 = measurement 1, 2–4 days following the onset of bleeding), the mid-follicular phase (M2 = measurement 2, 7–11 days following the onset of bleeding), the ovulatory phase (M3 = measurement 3, 1–2 days after positive urinary LH surge test, which may indicate ovulation) and the mid-luteal phase (M4 = measurement 4, 5–7 days after a positive urinary LH surge test). M1, M3 and M4 were defined according to the criteria published by Elliott-Sale et al. [18] while the time point for the second blood sampling in the present study (M2, mid-FP) was defined as the start of menses + 7–11 days. In IUD, blood samples were collected at four time points and measurements were arranged afterwards based on bleeding and/or measured hormonal values. From the IUD group, eight participants had bleeding/spotting during the study. Bleeding was defined as M1, however, if bleeding was not detected in IUD during the sample collection, M1 was defined according to following criteria: sample with lowest E2 concentration after sample with highest P4 concentration (*n* = 6) or sample after highest P4 concentration (*n* = 2) or sample with lowest E2 concentration if there were no significant changes in P4 (*n* = 2). Measurements were separated by approximately 7 days (range 5–9 days). In CHC, blood samples were collected at the end of inactive (i.e. pill/ring-free or placebo) phase (M1 = 25–28 days from the beginning of the active i.e. pill-consuming/ring-using phase), at the beginning of the active phase (M2 = 4–10 days from the beginning of the active phase), at the end of the active phase (M3 = 13–20 days from the beginning of the active phase), and at the beginning of the inactive phase (M4 = 22–24 days from the beginning of the active phase). An overview of the testing is presented in Fig. 1.

### Blood samples

To minimize possible diurnal variations in serum androgen concentrations, venous blood samples for hormone analyses were standardized and collected between 07.00 and 10.00 am in the morning after 10–12 h of overnight fasting, abstaining 12 h from caffeine and alcohol and 24 h from strenuous exercise [37]. Samples were taken from the antecubital vein into serum tubes (9 ml Venosafe, Terumo Medical Co., Belgium or Vacuette, Greiner Bio One International GmbH, Kremsmünster, Austria) and EDTA tubes (6 ml Venosafe EDTA Tubes, Terumo Medical Co., Belgium). Each participant’s basic blood count (analyzed from whole blood samples in the EDTA tubes by Sysmex KX-21 N, Kobe, Japan) was immediately analyzed for hematocrit (HCT).

The whole blood in the serum tubes for hormone analysis was stored at room temperature for 30 min, after which it was centrifuged for 10 min at 2000 × *g* (Venosafe tubes) or at 2245 × *g* (Vacuette tubes) and a refrigerated temperature of + 4 °C (Megafuge 1.0 R, Heraeus, Germany). The serum was separated and immediately frozen at −80 °C or first at −20 °C and later stored at −80 °C until the final analysis. Hormone analyses of E2, P4, LH, follicle-stimulating hormone (FSH), tT, dehydroepiandrosterone sulfate (DHEA-S), and SHBG were analyzed from serum using chemical luminescence techniques by Immulite 2000 XPi immunoassay system (Siemens Healthcare Diagnostics, Camberley, United Kingdom). fT and DHEA were analyzed using hormone specific immunoassay kits (Human testosterone, free ELISA, Biovendor or Testosterone free ELISA, Demeditec; DHEA ELISA, Demeditec) by Dynex Ds 2 ELISA processing System (Dynex Technologies, Chantilly, VA, USA). The analytical sensitivity was 55.0 pmol·L^−1^ for E2, 0.3 nmol·L^−1^ for P4, 0.05 IU·L^−1^ for LH, 0.1 IU·L^−1^ for FSH, 0.5 nmol·L^−1^ for tT, 0.06 pmol·L^−1^ (Biovendor) and 0.14 pmol·L^−1^ (Demeditec) for fT, 0.24 nmol·L^−1^ for DHEA, 0.08 µmol·L^−1^ for DHEA-S and 0.02 nmol·L^−1^ for SHBG. Inter-assay coefficients of variation were 9.7% and 15.2% for E2, 12.2% and 13.4% for P4, 10.7% and 8.2% for LH, 5.4% and 5.0% for FSH, 7.5% for tT, 12.4% for fT, 6.9% for DHEA, 9.8% and 9.2% for DHEA-S and 7.6% and 6.8% for SHBG in *MEndEx* and *ReWo* studies, respectively. Results of tT and fT were deleted from final data for one participant from NM due to laboratory error.

### LH surge test

LH surge, indicating ovulation, was assessed using a urinary LH test (Dipro, LH Ovulation Strip, Aidian Oy, Finland or Sofi, Finland) completed by the participants, at home, according to manufacturer’s instructions. The duration of previous MCs was used in combination with the LH surge test to determine the timing of M3 and M4 in the NM. Participants were asked not to perform the test using the first urine of the day, to avoid drinking excessive amounts of fluids for 2 h before the test, and to repeat the test at the same time every day. The blood sample for M3 was collected 1 to 2 days after a positive urinary LH surge test.

### Body mass and height

Anthropometric measurements were completed in the morning after 10–12 h of fasting at the beginning of the study period. Body mass was measured using calibrated scale. Participant height was measured with a wall-mounted stadiometer. To avoid the potential to influence eating and physical activity behaviors, participants were not given feedback regarding their body mass results until all the four measurement sessions were completed. BMI was calculated as weight divided by the square of height.

### Statistical analysis

Data were analyzed using IBM SPSS 28.0 (SPSS Inc., Chicago, IL). The effects of MC or HC phase on measured hormones, SHBG and HCT were analyzed separately for each of the three groups using generalized estimating equations (GEEs). GEEs were introduced by Liang and Zeger in 1986 as an extension of generalized linear models to analyze data collected in repeated measures designs [38]. The benefit of GEE model over repeated-measures ANOVA is that participants who have data available from only some of the data points are not lost as the GEE model utilizes information and considers correlation between repeated measurements for the same individual even when the data is incomplete. Due to the Covid19-pandemic some of the included participants were unable to provide data for all four time points and most of the participants using combined HCs from the *ReWo* study had only two time points measured as described previously [36] (see Table 3).

Linear GEE models with an unstructured working correlation matrix were constructed for each group to investigate differences between groups and changes in androgen hormones, SHBG and HCT across MC and HC phases measured at four time points. In addition, post hoc LSD-adjusted comparisons of time differences were performed. In the univariate models, the main factor was MC or HC phase. To investigate associations of changes in E2 and P4 with measured androgens and SHBG over MC and HC phases, androgen variables were adjusted with the change in E2 concentration (ΔE2) from M1 to M3 and the change in P4 concentration (ΔP4) from M2 to M4 in the multivariate models. Furthermore, the interactions between MC or HC phase and ΔE2 and ΔP4 were analyzed. In CHC, the associations between changes in concentrations of E2 and P4 and androgen variables were not analyzed, because there were no significant differences in concentrations of E2 and P4 between HC phases.

The participants from NM were divided for subgroup analysis based on P4 concentrations at M4 (> 16 nmol·L^−1^ and ≤ 16 nmol·L^−1^) to control for cycles that might have a deficient LP [16, 39]. One of the subjects from NM did not have data available from M4, thus this subject was included in the subgroup analysis of P4 concentrations ≤ 16 nmol·L^−1^. As participants in IUD had IUDs containing higher (Mirena; 52 mg) or lower (Jaydess; 13.5 mg and Kyleena; 19.5 mg) dose of progestin, we performed subgroup analysis. In IUD one participant had a high concentration of fT while another participant had a high concentration of DHEA. Therefore, we performed androgen and SHBG analyses without these individuals. This new analysis did not markedly affect the results reported in terms of androgen and SHBG levels. As such, we present the original analysis including these individuals. Regression coefficients (β), standard errors (SE), p-values and 95% confidence intervals (CI) are reported for each model. Statistical significance was set at *p* ≤ 0.05. Figures were prepared using GraphPad Prism 10.2.2 (GraphPad Software Inc., California, USA).

## Results

### Hormonal concentrations within groups

Serum concentrations of E2 and P4 reflected hormonal fluctuation expected during MC and HC phases (Table 3, Supplementary Figure S1, Additional File 1). In NM and IUD, the lowest mean concentrations of E2 were found at M1 and the highest at M3, whereas the lowest mean concentrations of P4 were found at M2 and highest at M4. LH surge was evident in all NM participants, but only 21 participants (58%) from NM had serum concentrations of P4 > 16 nmol·L^−1^ 5–7 days after a positive urinary LH surge test at M4, which typically corresponds with the mid-LP [18].

**Table 3:** Serum hormone and SHBG levels at different phases of menstrual cycle or hormonal contraceptive

NM	M1 (*n* = 33–35)	M2 (*n* = 31–33)	M3 (*n* = 29–30)	M4 (*n* = 33–35)
E2 (pmol·L^−1^)	164 (99–268)	330 (154–500)	525 (327–793)	562 (335–811)
P4 (nmol·L^−1^)	1.4 (1.0–1.8)	1.2 (0.8–1.4)	2.8 (1.7–5.9)	17.5 (6.7–24.5)
LH (IU·L^−1^)	5.4 (3.8–8.4)	6.3 (4.6–9.4)	11.8 (7.7–19.7)	5.5 (3.5–8.7)
FSH (IU·L^−1^)	6.8 (4.5–8.7)	6.3 (5.2–9.4)	6.8 (5.0–9.9)	2.8 (2.2–5.8)
tT (nmol·L^−1^)	0.8 (0.4–1.0)	0.7 (0.4–1.0)	1.0 (0.5–1.3)	0.9 (0.5–1.1)
fT (pmol·L^−1^)	6.6 (4.3–9.7)	8.4 (5.7–10.8)	9.4 (5.5–15.1)	8.0 (4.9–11.2)
DHEA (nmol·L^−1^)	45.5 (28.9–63.4)	48.1 (35.1–64.3)	53.5 (31.3–72.9)	45.9 (35.6–67.1)
DHEA-S (µmol·L^−1^)	5.5 (3.2–6.5)	5.6 (3.8–7.3)	4.9 (3.5–6.7)	5.3 (3.5–6.6)
SHBG (nmol·L^−1^)	60 (49–71)	59 (44–69)	61 (50–70)	60 (50–69)

IUD	**M1 (** ***n*** ** = 12)**	**M2 (** ***n*** ** = 12)**	**M3 (** ***n*** ** = 12)**	**M4 (** ***n*** ** = 12)**
E2 (pmol·L^−1^)	104 (74–145)	157 (108–303)	435 (208–1153)	392 (150–518)
P4 (nmol·L^−1^)	1.2 (0.9–1.9)	1.1 (0.7–1.5)	2.4 (0.9–3.8)	16.9 (1.9–23.8)
LH (IU·L^−1^)	6.2 (2.7–7.8)	8.0 (5.8–12.2)	12.1 (4.8–22.6)	6.3 (3.9–8.3)
FSH (IU·L^−1^)	5.8 (4.4–6.8)	7.6 (7.0–8.0)	7.3 (7.0–8.4)	3.9 (3.4–7.4)
tT (nmol·L^−1^)	0.5 (0.2–0.7)	0.6 (0.3–1.1)	0.8 (0.5–1.1)	0.6 (0.4–1.0)
fT (pmol·L^−1^)	6.3 (4.1–8.0)	6.8 (4.6–8.0)	6.9 (5.0–9.9)	5.3 (4.2–7.8)
DHEA (nmol·L^−1^)	37.5 (27.6–69.9)	44.7 (31.1–62.3)	43.9 (25.7–65.9)	31.9 (23.8–66.1)
DHEA-S (µmol·L^−1^)	4.4 (3.5–6.1)	4.2 (3.3–6.5)	4.2 (3.2–6.5)	3.9 (3.2–7.8)
SHBG (nmol·L^−1^)	44.4 (40.5–65.6)	42.7 (35.3–57.8)	47.8 (37.1–54.4)	45.2 (37.4–67.1)

CHC	**M1 (** *n* ** = 20)**	**M2 (** *n* ** = 16–18)**	**M3 (** *n* ** = 19–21)**	**M4 (** *n* ** = 14–15)**
E2 (pmol·L^−1^)	129 (66–261)	145 (92–206)	112 (60–186)	139 (94–254)
P4 (nmol·L^−1^)	0.9 (0.7–1.5)	1.2 (0.8–1.6)	1.0 (0.6–1.3)	1.0 (0.8–1.3)
LH (IU·L^−1^)	3.8 (0.7–6.5)	3.0 (0.3–6.2)	0.8 (0.2–2.8)	2.0 (0.3–4.5)
FSH (IU·L^−1^)	4.8 (1.2–6.2)	2.0 (0.9–3.6)	1.6 (0.4–3.0)	3.6 (0.4–6.0)
tT (nmol·L^−1^)	0.5 (0.4–1.4)	0.8 (0.2–1.1)	0.6 (0.3–0.9)	0.8 (0.3–1.3)
fT (pmol·L^−1^)	4.6 (3.1–7.8)	4.3 (2.8–8.0)	4.2 (2.9–5.0)	5.1 (3.0–8.2)
DHEA (nmol·L^−1^)	34.2 (20.6–53.4)	36.6 (27.1–50.3)	30.3 (18.4–47.6)	35.1 (24.8–60.0)
DHEA-S (µmol·L^−1^)	3.7 (2.9–5.3)	3.5 (2.8–5.4)	3.5 (2.3–4.7)	3.9 (2.7–5.0)
SHBG (nmol·L^−1^)	153 (99–225)	193 (122–245)	187 (117–253)	245 (156–270)

Values are medians (interquartile range)

*NM* Naturally menstruating females (M1 = bleeding, M2 = mid-follicular phase, M3 = ovulatory phase, M4 = mid-luteal phase), *IUD* Hormonal intrauterine device using females (M1 = bleeding or lowest E2 concentration and/or sample after highest P4 concentration, M2 = M1 + 7 days, M3 = M1 + 14 days, M4 = M1 + 21 days), *CHC* Combined hormonal contraceptive using females (M1 = end of inactive phase, M2 = beginning of active phase, M3 = end of active phase, M4 = beginning of inactive phase), *E2* Estradiol, *P4* Progesterone, *LH* Luteinizing hormone, *FSH* Follicle-stimulating hormone, *tT* Total testosterone, *fT* Free testosterone, *DHEA* Dehydroepiandrosterone, *DHEA-S* Dehydroepiandrosterone sulfate, *SHBG* Sex hormone-binding globulin

**Fig. 2:**
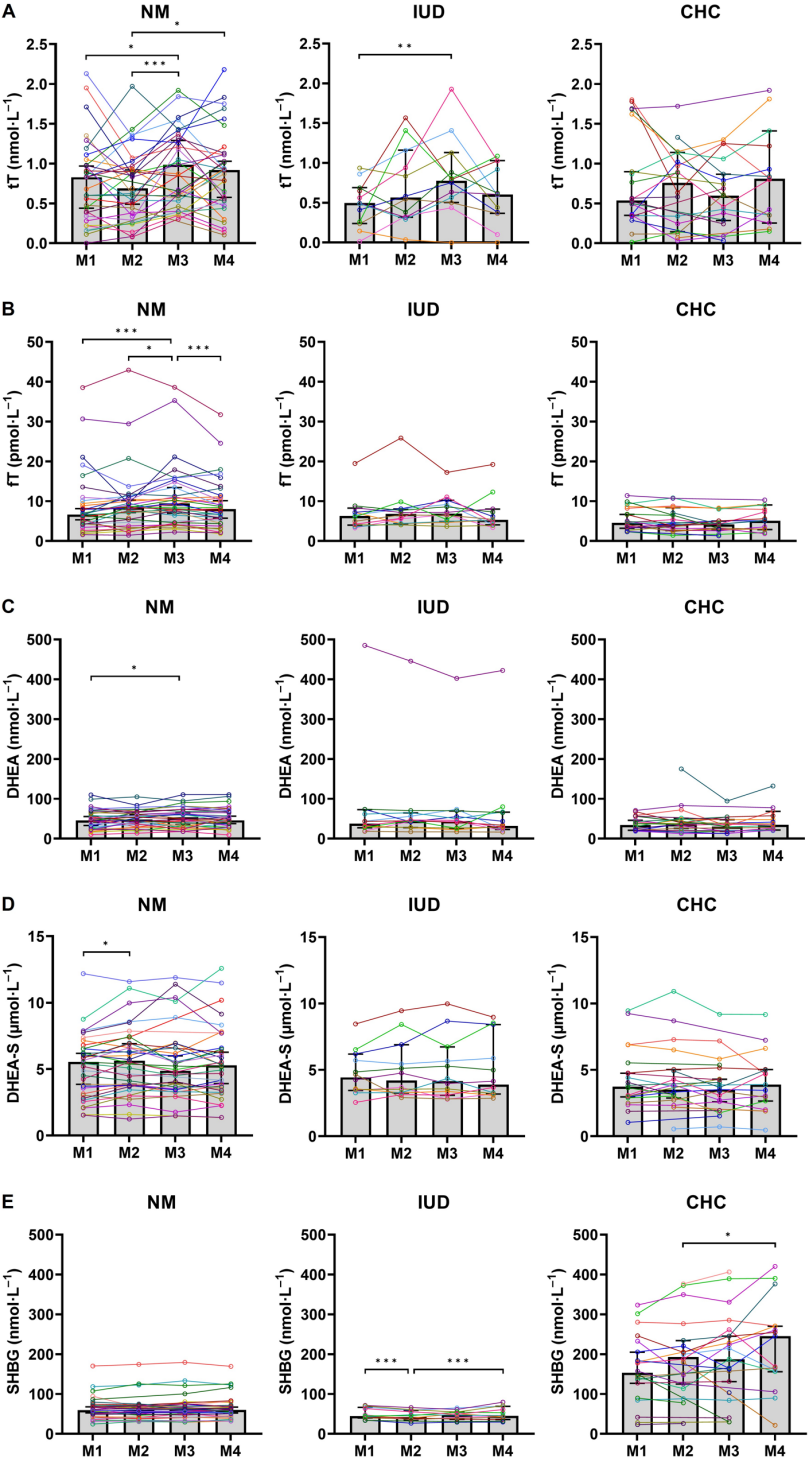
Changes in concentrations of androgens and SHBG during menstrual cycle and hormonal contraceptive phases. Median, 95% confidence interval, p-values of post hoc analyses, and individual hormone profiles of A) total testosterone (tT), B) free testosterone (fT), C) dehydroepiandrosterone (DHEA), D) dehydroepiandrosterone sulfate (DHEA-S), and E) sex hormone-binding globulin (SHBG) for naturally menstruating females (NM; M1 = bleeding, M2 = mid-follicular phase, M3 = ovulatory phase, M4 = mid-luteal phase), hormonal intrauterine device using females (IUD; M1 = bleeding or lowest estradiol concentration and/or sample after highest progesterone concentration, M2 = M1 + 7 days, M3 = M1 + 14 days, M4 = M1 + 21 days) and combined hormonal contraceptive using females (CHC; M1 = end of inactive phase, M2 = beginning of active phase, M3 = end of active phase, M4 = beginning of inactive phase). Significant difference * = *p* ≤ 0.05, ** = *p* < 0.01, *** = *p* < 0.001

In the analyses of androgens, the univariate GEE models revealed significant β-values between M1 and other MC and HC phases, which reflected results found in the post hoc analyses (Fig. 2). In NM, serum concentrations of tT, fT, and DHEA were highest at M3 being significantly different from M1 (Table 4). The post hoc analysis revealed that serum concentrations of tT were also significantly lower at M2 compared to M3 (*p* < 0.001) and M4 (*p* = 0.010) in NM. In the post hoc analysis, concentrations of fT were significantly higher at M3 compared to M2 (*p* = 0.020) and M4 (*p* < 0.001) in NM. Concentrations of DHEA-S fluctuated significantly in NM, being significantly higher at M2 than at M1. Concentrations of SHBG remained unchanged throughout the MC in NM.

**Table 4:** Changes in androgen and SHBG levels and associations with changes in estradiol and progesterone concentrations in NM

	Univariate model			Multivariate model 1				Multivariate model 2			
	M2 vs. M1	M3 vs. M1	M4 vs. M1	M2 vs. M1	M3 vs. M1	M4 vs. M1	ΔE2	M2 vs. M1	M3 vs. M1	M4 vs. M1	ΔP4
**tT (nmol·L** ^**−1**^ **)**											
**β(SE)**	−0.06 (0.07)	**0.16 (0.08)**	0.12 (0.08)	−0.08 (0.08)	0.15 (0.09)	0.12 (0.08)	0.00 (0.00)	−0.07 (0.07)	0.16 (0.09)	0.12 (0.08)	0.01 (0.01)
**95% CI**	−0.20, 0.08	**0.00**,** 0.32**	−0.04, 0.27	−0.23, 0.08	−0.02, 0.32	−0.04, 0.27	0.00, 0.00	−0.21, 0.08	−0.01, 0.33	−0.04, 0.28	−0.01, 0.02
** P**	0.390	**0.050**	0.134	0.342	0.082	0.147	0.239	0.383	0.060	0.151	0.348
**fT (pmol·L** ^**−1**^ **)**											
**β(SE)**	0.48 (0.57)	**1.85 (0.41)**	0.14 (0.47)	0.49 (0.64)	**1.85 (0.43)**	0.10 (0.52)	−0.00 (0.00)	0.52 (0.58)	**1.97 (0.43)**	0.16 (0.51)	0.17 (0.17)
** 95% CI**	**−**0.65, 1.60	**1.04**,** 2.66**	−0.77, 1.06	−0.77, 1.74	**1.01**,** 2.68**	−0.91, 1.12	−0.01, 0.00	−0.62, 1.66	**1.14**,** 2.80**	−0.83, 1.16	−0.16, 0.50
** P**	0.403	**< 0.001**	0.761	0.448	**< 0.001**	0.844	0.397	0.369	**< 0.001**	0.749	0.309
**DHEA (nmol·L** ^**−1**^ **)**											
** β(SE)**	2.03 (2.29)	**5.29 (2.64)**	4.16 (2.51)	2.40 (2.51)	**5.89 (2.75)**	4.52 (2.59)	0.00 (0.01)	1.63 (2.42)	5.17 (2.76)	3.91 (2.57)	**1.22 (0.53)**
** 95% CI**	−2.45, 6.51	**0.12**,** 10.47**	−0.75, 9.07	−2.53, 7.32	**0.51**,** 11.28**	−0.55, 9.59	−0.02, 0.02	−3.11, 6.37	−0.24, 10.58	−1.13, 8.95	**0.18**,** 2.26**
** P**	0.374	**0.045**	0.097	0.340	**0.032**	0.081	0.971	0.499	0.061	0.128	**0.021**
**DHEA-S (µmol·L** ^**−1**^ **)**											
** β(SE)**	**0.32 (0.13)**	0.18 (0.20)	0.24 (0.16)	**0.34 (0.14)**	0.23 (0.20)	0.16 (0.16)	0.00 (0.00)	**0.36 (0.13)**	0.20 (0.20)	0.29 (0.18)	0.02 (0.04)
** 95% CI**	**0.07**,** 0.57**	−0.21, 0.58	−0.08, 0.56	**0.06**,** 0.62**	−0.15, 0.62	−0.15, 0.47	0.00, 0.00	**0.10**,** 0.61**	−0.19, 0.59	−0.06, 0.65	−0.05, 0.09
**P**	**0.012**	0.355	0.144	**0.017**	0.238	0.317	0.239	**0.007**	0.314	0.105	0.653
**SHBG (nmol·L** ^**−1**^ **)**											
** β(SE)**	0.38 (1.55)	1.73 (1.54)	2.20 (1.61)	1.08 (1.23)	2.40 (1.53)	2.20 (1.62)	0.03 (0.02)	−0.36 (1.38)	1.81 (1.55)	1.17 (1.33)	−0.11 (0.42)
** 95% CI**	−2.66, 3.42	−1.29, 4.75	−0.95, 5.35	−1.33, 3.49	−0.60, 5.39	−0.97, 5.37	0.00, 0.07	−3.06, 2.35	−1.23, 4.85	−1.43, 3.77	−0.93, 0.71
** P**	0.805	0.261	0.172	0.379	0.117	0.174	0.078	0.796	0.243	0.377	0.795

Significant findings are denoted in bold

Values are presented as regression coefficients (β), standard errors (SE) and 95% confidence intervals (CI)

*tT* Total testosterone, *fT* Free testosterone, *DHEA* Dehydroepiandrosterone, *DHEA-S* Dehydroepiandrosterone sulfate, *SHBG* Sex hormone-binding globulin, *NM* Naturally menstruating females, *M1* Bleeding, *M2* Mid-follicular phase, *M3* Ovulatory phase, *M4* Mid-luteal phase, *ΔE2* Change in estradiol concentration from M1 to M3, *ΔP4* Change in progesterone concentration from M2 to M4

In IUD, serum concentrations of tT were highest at M3 being significantly different from M1 (Table 5). There were no significant differences in concentrations of fT, DHEA or DHEA-S in IUD. In IUD, concentrations of SHBG were lowest at M2 being significantly different from M1 and from M4 (*p* < 0.001). Based on the subgroup analysis (Table 6), levels of tT remained significantly higher at M3 compared to M1 in both subgroups, but in participants using Jaydess or Kyleena, concentrations of tT were also significantly higher at M3 compared to M2 (*p* < 0.001) and M4 (*p* = 0.010). In addition, the highest concentrations of fT were observed at M3 being significantly different from M1, M2 (*p* = 0.030) and M4 (*p* = 0.011), while levels of DHEA were significantly lower at M4 compared to M1 only in participants using Jaydess or Kyleena. Changes in serum concentrations of E2, P4, LH and FSH in subgroups are presented in Supplementary Table S5, Additional File 5.

**Table 5:** Changes in androgen and SHBG levels and associations with changes in estradiol and progesterone concentrations in IUD

	Univariate model			Multivariate model 1				Multivariate model 2			
	M2 vs. M1	M3 vs. M1	M4 vs. M1	M2 vs. M1	M3 vs. M1	M4 vs. M1	ΔE2	M2 vs. M1	M3 vs. M1	M4 vs. M1	ΔP4
**tT (nmol·L** ^**−1**^ **)**											
** β(SE)**	0.22 (0.12)	**0.34 (0.10)**	0.12 (0.10)								
** 95% CI**	−0.02, 0.46	**0.14**,** 0.55**	−0.08, 0.32								
**P**	0.076	**0.001**	0.234								
**fT (pmol·L** ^**−1**^ **)**											
** β(SE)**	1.12 (0.67)	0.96 (0.75)	0.10 (0.62)	1.12 (0.67)	0.96 (0.75)	0.10 (0.62)	0.00 (0.00)				
**95% CI**	−0.18, 2.43	−0.50, 2.43	−1.11, 1.31	−0.18, 2.43	−0.50, 2.43	−1.11, 1.31	0.00, 0.01				
**P**	0.091	0.197	0.869	0.091	0.197	0.869	0.782				
**DHEA (nmol·L** ^**−1**^ **)**											
** β(SE)**	−0.60 (5.14)	−5.54 (7.76)	−6.65 (7.30)	−0.60 (5.14)	−5.54 (7.76)	−6.65 (7.30)	−0.07 (0.06)	−0.60 (5.14)	−5.54 (7.76)	−6.65 (7.30)	0.49 (0.85)
** 95% CI**	−10.68, 9.47	−20.76, 9.68	−20.95, 7.65	−10.68, 9.47	−20.76, 9.68	−20.95, 7.65	−0.20, 0.05	−10.68, 9.47	−20.76, 9.68	−20.95, 7.65	−1.17, 2.16
** P**	0.906	0.476	0.362	0.906	0.476	0.362	0.251	0.906	0.476	0.362	0.561
**DHEA-S (µmol·L** ^**−1**^ **)**											
**β(SE)**	0.24 (0.24)	0.28 (0.30)	0.27 (0.30)	0.24 (0.24)	0.28 (0.30)	0.27 (0.30)	0.00 (0.00)	0.24 (0.24)	0.28 (0.30)	0.27 (0.30)	0.02 (0.05)
** 95% CI**	−0.23, 0.71	−0.32, 0.87	−0.33, 0.86	−0.23, 0.71	−0.32, 0.87	−0.33, 0.86	0.00, 0.00	−0.23, 0.71	−0.32, 0.87	−0.33, 0.86	−0.08, 0.12
**P**	0.311	0.362	0.377	0.311	0.362	0.377	0.815	0.311	0.362	0.377	0.696
**SHBG (nmol·L** ^**−1**^ **)**											
** β(SE)**	**−4.73 (1.36)**	−3.56 (2.10)	0.49 (1.27)	**−4.73 (1.36)**	−3.56 (2.10)	0.49 (1.27)	0.00 (0.01)	**−4.73 (1.36)**	−3.56 (2.10)	0.49 (1.27)	0.12 (0.31)
** 95% CI**	**−7.40**,** −2.05**	−7.67, 0.55	−2.00, 2.98	**−7.40**,** −2.05**	−7.67, 0.55	−2.00, 2.98	−0.01, 0.02	**−7.40**,** −2.05**	−7.67, 0.55	−2.00, 2.98	−0.50, 0.73
**P**	**< 0.001**	0.090	0.699	**< 0.001**	0.090	0.699	0.751	**< 0.001**	0.090	0.699	0.714

Significant findings are denoted in bold. Where the validity of the model fit was uncertain, adjusted results are not presented

Values are presented as regression coefficients (β), standard errors (SE) and 95% confidence intervals (CI)

*tT* Total testosterone, *fT* Free testosterone, *DHEA* Dehydroepiandrosterone, *DHEA-S* Dehydroepiandrosterone sulfate, *SHBG* Sex hormone-binding globulin, *IUD* Hormonal intrauterine device using females, *M1* Bleeding or lowest estradiol concentration and/or sample after highest progesterone concentration, *M2* M1 + 7 days, *M3* M1 + 14 days, *M4* M1 + 21 days, *ΔE2* Change in estradiol concentration from M1 to M3, *ΔP4* change in progesterone concentration from M2 to M4

**Table 6:** Changes in androgen and SHBG levels in participants using Jaydess or Kyleena and Mirena intrauterine device

	Jaydess & Kyleena (*n* = 6)			Mirena (*n* = 6)		
	M2 vs. M1	M3 vs. M1	M4 vs. M1	M2 vs. M1	M3 vs. M1	M4 vs. M1
**tT (nmol·L** ^**−1**^ **)**						
** β(SE)**	0.04 (0.10)	**0.45 (0.18)**	0.01 (0.14)	**0.40 (0.20)**	**0.23 (0.09)**	0.23 (0.13)
** 95% CI**	−0.17, 0.25	**0.11**,** 0.80**	−0.27, 0.28	**0.01**,** 0.79**	**0.05**,** 0.41**	−0.02, 0.48
** P**	0.701	**0.011**	0.961	**0.045**	**0.012**	0.065
**fT (pmol·L** ^**−1**^ **)**						
** β(SE)**	0.17 (0.11)	**0.64 (0.29)**	−0.15 (0.16)	0.48 (0.36)	−0.08 (0.25)	0.21 (0.30)
** 95% CI**	−0.04, 0.38)	**0.08**,** 1.20**	−0.46, 0.16	−0.22, 1.18	−0.56, 0.40	−0.39, 0.80
** P**	0.118	**0.026**	0.343	0.179	0.738	0.493
**DHEA (nmol·L** ^**−1**^ **)**						
** β(SE)**	−0.25 (1.77)	0.37 (1.89)	**−2.81 (1.40)**	−0.09 (2.37)	−3.57 (3.90)	−1.02 (3.94)
**95% CI**	−3.73, 3.23	−3.34, 4.09	**−5.55**,** −0.07**	−4.75, 4.56	−11.20, 4.07	−8.74, 6.69
**P**	0.886	0.844	**0.044**	0.968	0.360	0.795
**DHEA-S (µmol·L** ^**−1**^ **)**						
**β(SE)**	0.22 (0.14)	**0.76 (0.35)**	0.56 (0.35)	0.27 (0.46)	−0.21 (0.41)	−0.02 (0.47)
**95% CI**	−0.05, 0.49	**0.08**,** 1.44**	−0.13, 1.25	−0.64, 1.17	−1.01, 0.60	−0.93, 0.89
** P**	0.106	**0.029**	0.114	0.565	0.614	0.966
**SHBG (nmol·L** ^**−1**^ **)**						
** β(SE)**	**−4.85 (2.13)**	**−5.80 (2.80)**	−0.33 (1.20)	**−4.60 (1.70)**	−1.32 (2.84)	1.32 (2.19)
** 95% CI**	**−9.02**,** −0.68**	**−11.29**,** −0.31**	−2,68, 2.01	**−7.93**,** −1.27**	−6.89, 4.25	−2.98, 5.62
** P**	**0.023**	**0.039**	0.780	**0.007**	0.643	0.548

Significant findings are denoted in bold

Values are presented as regression coefficients (β), standard errors (SE) and 95% confidence intervals (CI)

*tT* Total testosterone, *fT* Free testosterone, *DHEA* Dehydroepiandrosterone, *DHEA-S* Dehydroepiandrosterone sulfate, *SHBG* Sex hormone-binding globulin, *M1* Bleeding or lowest estradiol concentration and/or sample after highest progesterone concentration, *M2* M1 + 7 days, *M3* M1 + 14 days, *M4* M1 + 21 days

Concentrations of E2, P4, tT, fT, DHEA, and DHEA-S remained unchanged between all measurement points in CHC (Table 7). In CHC, concentrations of LH were significantly lower at M3 and M4 and FSH at M2 and M3 compared to M1 (See Supplementary Table S1, Additional File 1). In post hoc analysis, concentrations of FSH were significantly lower also at M3 compared to M4 (*p* = 0.014). Concentrations of SHBG were significantly lower at M2 compared to M4 (*p* = 0.038) in CHC.

**Table 7:** Changes in androgen and SHBG levels for combined hormonal contraceptive using females

		M2 vs. M1	M3 vs. M1	M4 vs. M1
**tT (nmol·L** ^**−1**^ **)**				
**β(SE)**		−0.06 (0.13)	0.00 (0.10)	0.04 (0.11)
** 95% CI**		−0.32, 0.20	−0.20, 0.21	−0.18, 0.25
**P**		0.641	0.980	0.749
**fT (pmol·L** ^**−1**^ **)**				
**β(SE)**		−0.29 (0.39)	−0.53 (0.31)	−0.10 (0.39)
**95% CI**		−1.05, 0.47	−1.05, 0.47	−0.86, 0.66
** P**		0.456	0.456	0.796
**DHEA (nmol·L** ^**−1**^ **)**				
** β(SE)**		5.11 (4.53)	−1.77 (3.79)	1.03 (2.44)
**95% CI**		−3.76, 13.98	−9.19, 5.65	−3.76, 5.82
**P**		0.259	0.640	0.673
**DHEA-S (µmol·L** ^**−1**^ **)**				
** β(SE)**		0.22 (0.17)	−0.13 (0.16)	−0.18 (0.26)
**95% CI**		−0.12, 0.55	−0.44, 0.18	−0.69, 0.33
** P**		0.204	0.412	0.486
**SHBG (nmol·L** ^**−1**^ **)**				
**β(SE)**		−5.02 (12.66)	3.27 (13.56)	19.87 (13.05)
** 95% CI**		−29.83, 19.79	−23.30, 29.84	−5.71, 45.44
** P**		0.692	0.809	0.128

Significant findings are denoted in bold

Values are presented as regression coefficients (β), standard errors (SE) and 95% confidence intervals (CI)

*tT* Total testosterone, *fT* Free testosterone, *DHEA* Dehydroepiandrosterone, *DHEA-S* Dehydroepiandrosterone sulfate, *SHBG* Sex hormone-binding globulin, *M1* End of inactive phase, *M2* Beginning of active phase, *M3* End of active phase, *M4* Beginning of inactive phase

Mean and standard deviation as well as change of HCT at the different phases of MC or HC phases are presented in Supplementary Table S2 and S3, Additional File 2.

### Association of changes in female sex hormones with androgens

The associations between the changes in concentrations of E2 and P4 with changes in concentrations of androgens (tT, fT, DHEA, DHEA-S) and SHBG are presented in Table 4 (NM) and Table 5 (IUD). In NM, when adjusted with ΔE2 (M1 vs. M3) or ΔP4 (M2 vs. M4) in the multivariate model 1 and 2, the difference in concentrations of tT between M1 and M3 was no longer significant. In addition, ΔP4 was significantly associated with DHEA in NM. In IUD, ΔE2 was not significantly associated with fT, DHEA, DHEA-S or SHBG in multivariate model 1. In IUD, the validity of the model fit was uncertain when adjusting tT with ΔE2 in multivariate model 1, so the results are not presented. In IUD, ΔP4 was not significantly associated with DHEA, DHEA-S, or SHBG in multivariate model 2, but the validity of the model fit was uncertain when adjusting tT and fT with ΔP4, so the results are not presented.

The interaction of HC phase and ΔE2 was associated with the concentrations of tT (β = 0.00, *p* = 0.002) in IUD. In addition, the interaction between HC phase and ΔP4 was significantly associated with concentrations of DHEA (β = −0.12, *p* = 0.013) and DHEA-S (β = 0.02, *p* = 0.010). The validity of the model fit was, however, uncertain for interaction of HC phase with both ΔE2 and ΔP4 in fT.

As only 58% of participants in NM had serum concentrations of P4 > 16 nmol·L^−1^ at M4, we performed a subgroup analysis by dividing participants from NM based on concentrations of P4 at M4 (P4 > 16 nmol·L^−1^ or ≤ 16 nmol·L^−1^). The subgroup analysis yielded distinct results, as less pronounced fluctuations were observed in the androgen levels between MC phases in participants who had P4 ≤ 16 nmol·L^−1^ (for subgroup analysis data, see Additional File 3).

### Hormonal concentrations between groups

There were significant differences in serum concentrations of E2, P4, LH, and FSH between all three groups (see Additional File 4). Concentrations of tT, DHEA and DHEA-S were higher at M3 in NM compared to M3 in CHC (β = 0.36, *p* = 0.005; β = 19.28, *p* = 0.002; β = 1.66, *p* = 0.011, respectively). In addition, concentrations of DHEA were higher in NM than in CHC at M1 (β = 11.02, *p* = 0.042). In NM, concentrations of tT were higher at M1 and M4 compared to IUD (β = 0.29, *p* = 0.014; β = 0.29, *p* = 0.028, respectively). Concentrations of fT were lower at all measurement points in CHC compared to NM (β = −3.76, *p* = 0.013; β = −4.61, *p* = 0.004; β = −7.25, *p* < 0.001; β = −3.67; *p* = 0.006). Concentrations of fT were lower at M3 in CHC compared to corresponding measurement point in IUD (β = −3.75 *p* < 0.001). SHBG was higher in CHC at all measurement points compared to NM (β = 98.72; β = 133.84; β = 128.15; β = 157.81; *p* < 0.001) and IUD (β = 112.40; β = 150.87; β = 147.85; β = 172.97, *p* < 0.001) whereas SHBG was lower in IUD at all measurement points compared to NM (β = −13.68, *p* = 0.022; β = −17.03, *p* = 0.005; β = −19.71; *p* = 0.002; β = −15.17, *p* = 0.21).

## Discussion

The aim of this study was to investigate the changes in serum concentrations of endogenous androgens and SHBG over different phases of the MC in physically active naturally menstruating females and in different phases of the HC in physically active females using hormonal intrauterine devices (IUDs) or combined HCs. In addition, we compared hormone concentrations between the groups. The primary finding was that concentrations of androgens fluctuated significantly across the MC and HC phases of hormonal IUD users and remained unchanged across the HC phases of combined HC using females. The secondary finding was that androgen levels differed between all three groups of physically active females.

### Hormonal differences within groups

While serum concentrations of E2 and P4 reflected the hormonal concentrations expected during the MC and HC phases, only 58% of participants in NM had concentrations of P4 > 16 nmol·L^−1^ in LP, despite a positive urinary LH surge tests. This could be due to missing peak P4 concentrations when scheduling measurements or could be indicative of luteal phase deficiency (LPD; short LP and suboptimal P4 concentration during LP [40]). Furthermore, it should be noted that a positive LH surge test is not always indicative of ovulation [41].

In NM, concentrations of tT peaked significantly at ovulatory phase, in line with findings of past research [19–22]. In addition, concentrations of fT and DHEA appeared to be significantly elevated near ovulation, as well as concentrations of DHEA-S at mid-FP (M2) compared to bleeding (M1), while concentrations of SHBG remained unchanged during the MC. It has been previously reported that exercise performance is trivially reduced during the early FP of the MC with the greatest difference in performance being in comparison to the late FP of the MC [15]. Increased concentrations of androgens during the FP near ovulation might be linked to performance benefits reported in the late FP. In addition, FP-based strength training is suggested to be beneficial for gaining muscle strength [42, 43]. It is well established that androgens influence muscle mass, adipose tissue distribution, and bone metabolism and are involved in a variety of other biochemical and physiological functions affecting female health [11]. As such, the fluctuations of these hormones during MC and HC might influence the variation observed in performance during MC [15] and between naturally menstruating females and females using HC [16].

While it has been demonstrated that androgen levels change significantly over the MC [19–22], we also observed significant changes in androgen concentrations over the HC phases in females using LNG-releasing IUDs. In IUD, there were significant changes in concentrations of tT and SHBG. Similar to NM, concentrations of tT were highest at mid-cycle. The contraceptive effect of hormonal IUDs is based on changes in the milieu of uterus (thinning of the endometrium, glandular atrophy, stromal decidualization), and cervix (modifying the viscosity of the cervical mucus) preventing sperm, ovum and embryo migration and implantation [44]. Serum concentrations of LNG released by IUDs are significantly lower compared to other progestin-only HCs, which is why ovulation usually occurs regularly depending on the dose in which the progestin is administered [45, 46]. Participants in IUD used devices containing LNG with a total content ranging from 13.5 mg to 52 mg, depending on the brand (Table 2). Variability in the dose of the progestin and therefore in HPO-axis suppression in progestin-only HC users may influence sex steroid profiles including androgens.

Females using combined HCs (CHC) had relatively stable androgen concentrations across the HC phases. In females using combined monophasic OCs, concentrations of ethinyl estradiol increase twofold from day 1 to day 21 during the active phase, while concentrations of progestin increase threefold reaching steady-state levels after about 8–11 days of active phase [47, 48]. In this study the end of the inactive phase (M1) was meant to capture the hormonal milieu of increasing endogenous hormones and low exogenous hormones, the beginning of the active phase (M2) increasing exogenous hormone levels, the end of the active phase (M3) highest exogenous hormone levels and low endogenous hormones and the beginning of the inactive phase (M4) low endogenous and exogenous hormone levels [18].

In CHC, concentrations of SHBG were significantly higher at the beginning of the inactive phase (M4) compared to the beginning of the active phase (M2). Concentrations of serum SHBG are reported to increase by about 1.5–1.6-fold, during the 21-day active phase of combined monophasic OCs, which is induced by the action of ethinyl estradiol [48]. Within 4 to 5 days after the last hormone-containing pill administration, concentrations of SHBG decrease [48]. In our data, the decrease of elevated SHBG levels was not significant during the inactive phase, but only at the beginning of the active phase (M2), after which concentrations started to significantly increase towards the beginning of the inactive phase (M4). This is likely, in part, due to variation in the length of the inactive phase in participants (from 4 to 7 days) and due to the different estrogens and their effects on pituitary downregulation and SHBG synthesis [49]. This might also explain the variability observed in concentrations of tT and DHEA-S, as different estrogen preparations have been shown to affect the concentrations of tT and adrenal androgens, such as DHEA-S, differently [50]. Concentrations of tT remained unchanged during the active phase, in line with previous findings [48].

Exogenous hormones are not consumed during the inactive period of HC use, which decreases their inhibitory effect on endogenous hormone secretion [27]. While Rechichi et al. [51] found that downregulated concentrations of endogenous E2 rise during the inactive phase, Elliott et al. [17] found no differences in concentrations of E2 or P4 between the active and inactive phase, in line with our findings. In addition, there were no differences in concentrations of androgens between HC phases. Instead, concentrations of FSH were significantly higher during the inactive phase compared to both measurements during the active phase (See Supplementary Figure S1, Additional File 1). The most sensitive indices of pituitary-ovarian suppression seem to be levels of FSH, since its secretion significantly increases during the inactive phase and when a pill is skipped or forgotten, levels of FSH markedly increase without changes in the plasma levels of LH or E2 [30].

The difference in concentrations of tT between M1 and M3 in NM appears to be explained by the change in E2 and P4 as the result was no longer significant after adjustment with ΔE2 (M1 vs. M3) and ΔP4 (M2 vs. M4). This might be caused by biosynthesis of T from P4 in the ovary via several conversion processes although biosynthesis of T does not proceed in an entirely linear fashion [52]. In IUD, the significant interaction between ΔE2 and HC phase in tT appear due to heterogeneity in tT concentrations within the IUD group from M1 to M2 and from M3 to M4.

### Hormonal concentrations between groups

CHC had significantly lower concentrations of tT, DHEA and DHEA-S at the end of the active phase (M3) compared to ovulatory phase (M3) in NM and higher SHBG concentrations at all measurement points compared to both NM and IUD. Concentrations of fT were lower at all measurement points in CHC compared to NM and at M3 compared to IUD. Combined OCs have previously been shown to influence androgen concentrations by significantly decreasing tT and fT levels, which could be explained by their tendency to increase SHBG levels [53], as T binds to this carrier protein [6]. Our findings indicate that combined HCs downregulate concentrations of tT, fT, DHEA, and DHEA-S. Since androgens are precursors for estrogens in the ovarian metabolism of steroids, androgen levels are affected by ovarian suppression due to combined HC use. While decrease in androgen concentrations may be a desired outcome in some combined HC users, the exercise implications for physically active individuals and athletes not seeking this outcome are not well known. Concentrations of tT did not differ at ovulatory phase in NM and at mid-cycle in IUD (M3) but were higher in NM at bleeding (M1) and mid-LP (M4). Concentrations of SHBG were lower in IUD in all measurement points compared to NM which is in line with previous studies showing that mean concentrations of SHBG decrease during the use of LNG-containing IUDs [28, 54] and that concentrations of SHBG correlate significantly with concentrations of LNG in IUD users [28, 54, 55]. LNG is mainly bound to SHBG and has a significant ability to displace T from SHBG.

### Strengths and limitations

The results of the present study might provide important information regarding the endogenous androgen milieu in physically active and healthy females. Nevertheless, there are several limitations that should be considered. First, it is important to consider the risk of type II statistical error in the present analyses, given that this was a secondary analysis from pooled data and the sample size was originally determined based on power calculations for the primary outcomes (physical performance) in the main studies. This, and relatively small number of participants, especially in the IUD group, indicate that results should be interpreted with caution. Second, immunoassays have been shown to be insufficiently reliable for investigating testosterone concentrations in females, in whom low androgen concentrations are expected [56]. Third, we were unable to analyze all of the main androgens such as A4. While T can be formed in peripheral tissues to some extent from DHEA, which we measured in this study, T is primarily converted peripherally from A4 [4].

The CHC included participants who were taking a variety of combined monophasic HCs including both pills and rings (8 different hormone contents and 14 brands) instead of one type/brand as might be considered optimal in this type of research [18]. The inactive phase ranging from 4 to 7 days may have influenced endogenous hormonal profiles, where suppression of ovarian activity is more pronounced with a shorter inactive (pill-free) phase [57]. In addition, the differences in potency and androgenicity of included formulations of combined hormonal contraceptives may have influenced outcomes [31, 58]. Participants who had IUDs containing different dose of progestin were combined into the same group, which might have affected the results, as release rates and serum levels of LNG produced by Jaydess and Kyleena are lower compared to Mirena [45, 46]. As a result of lower systemic exposure to LNG, Jaydess and Kyleena users tend to experience ovulation more regularly than Mirena users, suggesting a lower inhibition of HPO-axis [45, 46]. The subgroup analysis separating IUD by dose yielded somewhat different results, showing more pronounced fluctuations of androgens in Jaydess and Kyleena IUD users. However, the sample size here may be too small to meaningfully interpret these results. It should be noted, that by the time the 52 mg IUD has been in for five years or more, the daily release rate of LNG is approximately the same as in the initial phases of the 13.5 mg and 19.5 mg IUDs, as the daily amount of LNG released by the IUD changes with time [59].

The timing of blood sampling can be challenging in hormonal IUD users as they might not experience any bleeding that would indicate a new cycle. While hormonal IUDs can reduce or prevent bleeding, no difference has been found in concentrations of E2, P4 or progestin between those with regular bleeding and those without [60], suggesting that bleeding alone is not a predictor of ovarian function. Thus, measurements were arranged based on hormonal values (E2 and P4) in the absence of bleeding. A strength of this study is that in addition to information on bleeding and using urinary LH surge test to identify possible ovulation (only in NM) hormonal analysis was used to confirm expected cycle phases in both NM and IUD while hormonal analysis was also performed in CHC [18, 39]. At the time of data collection, visual interpretation of tests was an accepted method whereas digital tests are currently considered more accurate.

It is important to remember that measurements at one pre-defined time point may not adequately represent the day-to-day hormonal changes [61]. Furthermore, the MC can fluctuate between normal ovulatory cycles and the development of LPD and other menstrual disturbances in physically active females and athletes [62], thus it is possible that following several consecutive MC and HC phases would have revealed different results.

### Future perspectives

To our knowledge, this is the first study to report data on androgen concentrations in three distinct groups of physically active females that were either naturally menstruating, using a LNG-releasing IUD or combined HCs. Future studies should use LH surge test to determine the presence or absence of ovulation in progestin-only HC users while considering their hormonal profiles. Furthermore, the impact of the day-to-day fluctuations in sex hormones, also in androgens, needs to be better understood, as most of the existing MC or HC research has focused on measurements in pre-defined phases [61] and often only examining E2 and P4.

## Conclusions

In physically active females, female sex hormone and androgen concentrations fluctuate during the MC and HC phases of hormonal IUD users. In contrast, female sex hormone and androgen concentrations do not fluctuate in combined HC users. Our study suggests that differences in androgen levels between the MC phases might be linked to changes in E2 and P4 concentrations. Since endogenous hormonal milieu, including androgens, influence various physiological systems and may have an impact on training responses and adaptations, the influence of changes in androgen levels during the MC and HC phases should be considered in physiological research. Although the increases in tT, fT, and DHEA at ovulatory phase were statistically significant, it is unknown if these changes exert meaningful physiological effects. Investigating the concurrent changes in sex hormones, including androgens, in physically active females might improve our understanding of the mechanisms behind training adaptations.

## Supplementary Information

Additional file 1 – Changes in E2, P4, LH and FSH. Supplementary Table S1 Changes in E2, P4, LH and FSH levels for NM, IUD and CHC. Supplementary Figure S1 Changes in concentrations of E2, P4, LH and FSH during menstrual cycle and hormonal contraceptive phases. Median, 95% confidence intervals, p-values of post hoc analyses, and individual hormone profiles of (**A**) estradiol (E2), (**B**) progesterone (P4), (**C**) luteinizing hormone (LH), and (**D**) follicle-stimulating hormone (FSH) for naturally menstruating females (NM), hormonal intrauterine device using females (IUD), and combined hormonal contraceptive using females (CHC).

Additional file 2 – Changes in hematocrit. Supplementary table S2 Hematocrit at different phases of menstrual cycle or hormonal contraceptive. Supplementary Table S3 Changes in hematocrit for naturally menstruating females (NM), hormonal intrauterine device using females (IUD), and combined hormonal contraceptive using females (CHC).

Additional file 3 – Results of subgroup analysis. Supplementary table S4 Changes in hormone and SHBG levels based on concentrations of P4 at M4 in NM. Supplementary Figure S2 Changes in concentrations of hormones and SHBG based on P4 concentrations at M4 in NM. Median, 95% confidence interval, p-values of post hoc analyses and individual hormone profiles of (**A**) estradiol (E2), (**B**) progesterone (P4), (**C**) luteinizing hormone (LH), (**D**) follicle-stimulating hormone (FSH), (**E**) sex hormone-binding globulin (SHBG), (**F**) total testosterone (tT), (**G**) free testosterone (fT), (**H**) dehydroepiandrosterone (DHEA), and (**I**) dehydroepiandrosterone sulfate (DHEA-S) for naturally menstruating females (NM; M1 = bleeding, M2 = mid-follicular phase, M3 = ovulatory phase, M4 = mid-luteal phase).

Additional file 4 – Concentrations of E2, P4, LH and FSH between groups.

Additional file 5 – Changes in E2, P4, LH and FSH in subgroup analysis of IUD. Supplementary Table S5 Changes in E2, P4, LH and FSH levels in participants using Jaydess or Kyleena and Mirena intrauterine device.

## Data Availability

The data presented in this study are available from the corresponding author on reasonable request.

## Abbreviations

A4: Androstenedione
BMI: Body mass index
CHC: Combined hormonal contraceptive using females
CI: Confidence interval
DHEA: Dehydroepiandrosterone
DHEA-S: Dehydroepiandrosterone sulfate
E2: Estradiol
FP: Follicular phase
FSH: Follicle-stimulating hormone
fT: Free testosterone
GEE: Generalized estimating equation
HC: Hormonal contraceptive
HCT: Hematocrit
HPO: Hypothalamic-pituitary-ovarian
IUD: Hormonal intrauterine device using females
LH: Luteinizing hormone
LNG: Levonorgestrel
LP: Luteal phase
LPD: Luteal phase deficiency
LSD: Least significant difference
MC: Menstrual cycle
M1: Measurement 1
M2: Measurement 2
M3: Measurement 3
M4: Measurement 4
NM: Naturally menstruating females
OC: Oral contraceptive pill
P4: Progesterone
SD: Standard deviation
SE: Standard error
SHBG: Sex hormone-binding globulin
T: Testosterone
tT: Total testosterone
V̇O_2peak_: Peak oxygen uptake
β: Regression coefficient
