# Supplementary material for: Changes in androgen profile over the menstrual cycle and hormonal contraceptive phases in physically active females
Source: BMC Womens Health. 2026 Jan 27;26:118. doi: 10.1186/s12905-025-04253-6 (PMC12918223; doi:10.1186/s12905-025-04253-6)
Supplement: Supplementary file 1 — Additional file 1 – Changes in E2, P4, LH and FSH. Supplementary Table S1 Changes in E2, P4, LH and FSH levels for NM, IUD and CHC. Supplementary Figure S1 Changes in concentrations of E2, P4, LH and FSH during menstrual cycle and hormonal contraceptive phases. Median, 95% confidence intervals, p-values of post hoc analyses, and individual hormone profiles of (A) estradiol (E2), (B) progesterone (P4), (C) luteinizing hormone (LH), and (D) follicle-stimulating hormone (FSH) for naturally menstruating females (NM), hormonal intrauterine device using females (IUD), and combined hormonal contraceptive using females (CHC). [file 12905_2025_4253_MOESM1_ESM.docx]

**Additional File 1 – Changes in E2, P4, LH and FSH**

Changes in Androgen Profile Over the Menstrual Cycle and Hormonal Contraceptive Phases in Physically Active Females

Vera M. Salmi^1^*, Ritva S. Mikkonen^1^, Ida E. Löfberg^1^, Kelly L. McNulty^2^, Kirsty M. Hicks^2,3^, Anthony C. Hackney^4^, Johanna K. Ihalainen^1,5^

1. Faculty of Sport and Health Sciences, University of Jyväskylä, Jyväskylä, Finland
2. Department of Sport, Exercise and Rehabilitation, Faculty of Health and Life Sciences, Northumbria University, Newcastle-upon-Tyne, UK
3. Performance, Medical and Innovation Department, Washington Spirit Soccer Club, Washington DC, USA
4. Department of Exercise & Sport Science – Department of Nutrition, University of North Carolina, Chapel Hill, North Carolina, USA
5. Finnish Institute of High Performance Sport KIHU, Jyväskylä, Finland

**Supplementary Table S1** **Changes in** **E2, P4, LH and FSH levels for NM, IUD and CHC**

|  | **NM** | | |  | **IUD** | | |  | **CHC** | | |
| --- | --- | --- | --- | --- | --- | --- | --- | --- | --- | --- | --- |
|  | **M2 vs. M1** | **M3 vs. M1** | **M4 vs. M1** |  | **M2 vs. M1** | **M3 vs. M1** | **M4 vs. M1** |  | **M2 vs. M1** | **M3 vs. M1** | **M4 vs. M1** |
| **E2 (pmol·L^−1^)** |  |  |  |  |  |  |  |  |  |  |  |
| **β (SE)** | **182.06 (45.50)** | **414.56 (72.22)** | **369.77 (39.32)** |  | **132.40 (67.24)** | **506.68 (133.84)** | **264.26 (46.18)** |  | 23.88 (85.07) | −14.24 (50.87) | −82.64 (49.25) |
| **95% CI** | **92.89, 271.24** | **273.02, 556.11** | **292.71, 446.83** |  | **0.62, 264.18** | **244.34, 769.01** | **173.74, 354.78** |  | −142.86, 190.62 | −113.94, 85.47 | −179.18, 13.90 |
| **P** | **< 0.001** | **< 0.001** | **< 0.001** |  | **0.049** | **< 0.001** | **< 0.001** |  | 0.779 | 0.780 | 0.093 |
| **P4 (nmol·L^−1^)** |  |  |  |  |  |  |  |  |  |  |  |
| **β (SE)** | **−0.56 (0.21)** | **2.16 (0.51)** | **15.09 (1.78)** |  | −0.65 (0.46) | 3.07 (2.64) | **13.82 (3.63)** |  | 0.23 (0.16) | 0.31 (0.19) | 0.29 (0.21) |
| **95% CI** | **−0.98, −0.15** | **1.15, 3.16** | **11.60, 18.59** |  | −1.55, 0.24 | −2.09, 8.24 | **6.70, 20.94** |  | −0.08, 0.54 | −0.06, 0.67 | −0.13, 0.70 |
| **P** | **0.007** | **< 0.001** | **< 0.001** |  | 0.154 | 0.244 | **< 0.001** |  | 0.139 | 0.096 | 0.178 |
| **LH (IU·L^−1^)** |  |  |  |  |  |  |  |  |  |  |  |
| **β (SE)** | 0.58 (0.66) | **11.39 (3.57)** | 0.56 (0.94) |  | **4.98 (2.22)** | **8.29 (2.40)** | 1.38 (1.03) |  | −0.77 (0.46) | **−2.14 (0.82)** | **−1.49 (0.51)** |
| **95% CI** | −0.71, 1.88 | **4.41, 18.38** | −1.29, 2.41 |  | **0.63, 9.32** | **3.59, 13.00** | −0.64, 3.39 |  | −1.67, 0.13 | **−3.75, −0.54** | **−2.49, −0.49** |
| **P** | 0.378 | **0.001** | 0.556 |  | **0.025** | **< 0.001** | 0.181 |  | 0.095 | **0.009** | **0.003** |
| **FSH (IU·L^−1^)** |  |  |  |  |  |  |  |  |  |  |  |
| **β (SE)** | 0.60 (0.49) | 1.59 (0.82) | **−2.25 (0.51)** |  | **1.85 (0.51)** | **1.49 (0.75)** | −1.05 (0.87) |  | **−1.51 (0.59)** | **−1.95 (0.66)** | −0.98 (0.73) |
| **95% CI** | −0.36, 1.55 | −0.02, 3.21 | **−3.24, −1.25** |  | **0.86, 2.85** | **0.02, 2.97** | −2.75, 0.65 |  | **−2.66, −0.35** | **−2.66, −0.35** | −2.41, 0.46 |
| **P** | 0.221 | 0.054 | **< 0.001** |  | **< 0.001** | **0.048** | 0.225 |  | **0.010** | **0.003** | 0.182 |

Values are presented as regression coefficients (β), standard errors (SE) and 95% confidence intervals (CI). E2, estradiol; P4, progesterone; LH, luteinizing hormone; FSH, follicle-stimulating hormone; NM, naturally menstruating females (M1 = bleeding, M2 = mid-follicular phase, M3 = ovulatory phase, M4 = mid-luteal phase); IUD, hormonal intrauterine device using females (M1 = bleeding or lowest E2 concentration and/or sample after highest P4 concentration, M2 = M1 + 7 days, M3 = M1 + 14 days, M4 = M1 + 21 days); CHC, combined hormonal contraceptive using females (M1 = end of inactive phase, M2 = beginning of active phase, M3 = end of active phase, M4 = beginning of inactive phase). Significant findings are denoted in bold

**Supplementary Figure S1**
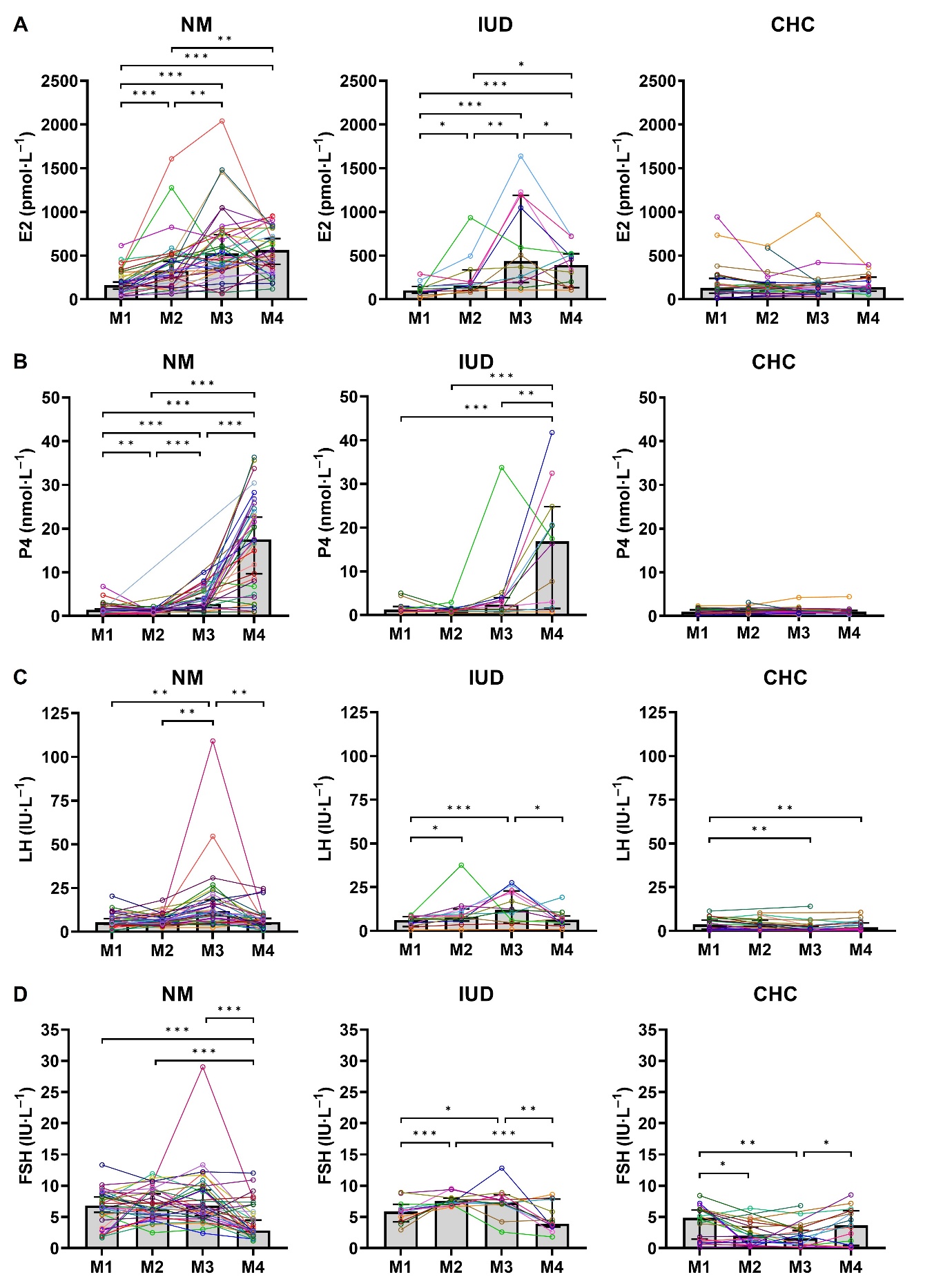


Changes in concentrations of E2, P4, LH and FSH during menstrual cycle and hormonal contraceptive phases. Median, 95% confidence intervals, p-values of post hoc analyses, and individual hormone profiles of **A)** estradiol (E2), **B)** progesterone (P4), **C)** luteinizing hormone (LH), and **D)** follicle-stimulating hormone (FSH) for naturally menstruating females (NM; M1 = bleeding, M2 = mid-follicular phase, M3 = ovulatory phase, M4 = mid-luteal phase), hormonal intrauterine device using females (IUD; M1 = bleeding or lowest E2 concentration and/or sample after highest P4 concentration, M2 = M1 + 7 days, M3 = M1 + 14 days, M4 = M1 + 21 days), and combined hormonal contraceptive using females (CHC; M1 = end of inactive phase, M2 = beginning of active phase, M3 = end of active phase, M4 = beginning of inactive phase). Significant difference * = *p* ≤ 0.05, ** = *p* < 0.01, *** = *p* < 0.001
