# Supplementary material for: Changes in androgen profile over the menstrual cycle and hormonal contraceptive phases in physically active females
Source: BMC Womens Health. 2026 Jan 27;26:118. doi: 10.1186/s12905-025-04253-6 (PMC12918223; doi:10.1186/s12905-025-04253-6)
Supplement: Supplementary file 2 — Additional file 2 – Changes in hematocrit. Supplementary table S2 Hematocrit at different phases of menstrual cycle or hormonal contraceptive. Supplementary Table S3 Changes in hematocrit for naturally menstruating females (NM), hormonal intrauterine device using females (IUD), and combined hormonal contraceptive using females (CHC). [file 12905_2025_4253_MOESM2_ESM.docx]

**Additional File 2 – Changes in hematocrit**

Changes in Androgen Profile Over the Menstrual Cycle and Hormonal Contraceptive Phases in Physically Active Females

Vera M. Salmi^1^*, Ritva S. Mikkonen^1^, Ida E. Löfberg^1^, Kelly L. McNulty^2^, Kirsty M. Hicks^2,3^, Anthony C. Hackney^4^, Johanna K. Ihalainen^1,5^

1. Faculty of Sport and Health Sciences, University of Jyväskylä, Jyväskylä, Finland
2. Department of Sport, Exercise and Rehabilitation, Faculty of Health and Life Sciences, Northumbria University, Newcastle-upon-Tyne, UK
3. Performance, Medical and Innovation Department, Washington Spirit Soccer Club, Washington DC, USA
4. Department of Exercise & Sport Science – Department of Nutrition, University of North Carolina, Chapel Hill, North Carolina, USA
5. Finnish Institute of High Performance Sport KIHU, Jyväskylä, Finland

**Supplementary Table S2 Hematocrit at different phases of menstrual cycle or hormonal contraceptive**

|  | **M1** | **M2** | **M3** | **M4** |
| --- | --- | --- | --- | --- |
| **NM** |  |  |  |  |
| HCT (%) | 40.7 ± 1.7 | 41.2 ± 1.9 | 40.6 ± 1.8 | 40.8 ± 2.0 |
| **IUD** |  |  |  |  |
| HCT (%) | 49.0 ± 25.3 | 48.4 ± 25.5 | 49.3 ± 24.6 | 47.6 ± 25.7 |
| **CHC** |  |  |  |  |
| HCT (%) | 42.0 ± 2.3 | 40.3 ± 2.5 | 40.5 ± 2.9 | 40.8 ± 2.9 |

Values are means ± SD. MC, menstrual cycle; HC, hormonal contraceptive; HCT, hematocrit; NM, naturally menstruating females (M1 = bleeding, M2 = mid-follicular phase, M3 = ovulatory phase, M4 = mid-luteal phase); IUD, hormonal intrauterine device using females (M1 = bleeding or lowest estradiol concentration and/or sample after highest progesterone concentration, M2 = M1 + 7 days, M3 = M1 + 14 days, M4 = M1 + 21 days); CHC, combined hormonal contraceptive using females (M1 = end of inactive phase, M2 = beginning of active phase, M3 = end of active phase, M4 = beginning of inactive phase)

**Supplementary Table S3 Changes in hematocrit for NM, IUD and CHC**

|  | **M2 vs. M1** | **M3 vs. M1** | **M4 vs. M1** |
| --- | --- | --- | --- |
| **NM** |  |  |  |
| **β (SE)** | **0.59 (0.29)** | −0.05 (0.28) | 0.11 (0.24) |
| **95% CI** | **0.02, 1.17** | −0.60, 0.49 | −0.35, 0.58 |
| **P** | **0.042** | 0.849 | 0.632 |
| **IUD** |  |  |  |
| **β (SE)** | −0.58 (0.61) | 0.25 (0.24) | **−1.42 (0.51)** |
| **95% CI** | −1.77, 0.61 | −0.22, 0.72 | **−2.41, −0.42** |
| **P** | 0.336 | 0.296 | **0.005** |
| **CHC** |  |  |  |
| **β (SE)** | −1.01 (0.54) | −0.91 (0.58) | −0.77 (0.52) |
| **95% CI** | −2.07, 0.05 | −2.04, 0.22 | −1.79, 0.26 |
| **P** | 0.062 | 0.116 | 0.142 |

Values are presented as regression coefficients (β), standard errors (SE) and 95% confidence intervals (CI). HCT, hematocrit; NM, naturally menstruating females (M1 = bleeding, M2 = mid-follicular phase, M3 = ovulatory phase, M4 = mid-luteal phase); IUD, hormonal intrauterine device using females (M1 = bleeding or lowest estradiol concentration and/or sample after highest progesterone concentration, M2 = M1 + 7 days, M3 = M1 + 14 days, M4 = M1 + 21 days); CHC, combined hormonal contraceptive using females (M1 = end of inactive phase, M2 = beginning of active phase, M3 = end of active phase, M4 = beginning of inactive phase). Significant findings are denoted in bold. In post hoc analysis, HCT was significantly lower at M4 compared to M3 (*p* = 0.002) and M2 (*p* = 0.024) in IUD
