# Supplementary material for: Changes in androgen profile over the menstrual cycle and hormonal contraceptive phases in physically active females
Source: BMC Womens Health. 2026 Jan 27;26:118. doi: 10.1186/s12905-025-04253-6 (PMC12918223; doi:10.1186/s12905-025-04253-6)
Supplement: Supplementary file 3 — Additional file 3 – Results of subgroup analysis. Supplementary table S4 Changes in hormone and SHBG levels based on concentrations of P4 at M4 in NM. Supplementary Figure S2 Changes in concentrations of hormones and SHBG based on P4 concentrations at M4 in NM. Median, 95% confidence interval, p-values of post hoc analyses and individual hormone profiles of (A) estradiol (E2), (B) progesterone (P4), (C) luteinizing hormone (LH), (D) follicle-stimulating hormone (FSH), (E) sex hormone-binding globulin (SHBG), (F) total testosterone (tT), (G) free testosterone (fT), (H) dehydroepiandrosterone (DHEA), and (I) dehydroepiandrosterone sulfate (DHEA-S) for naturally menstruating females (NM; M1 = bleeding, M2 = mid-follicular phase, M3 = ovulatory phase, M4 = mid-luteal phase). [file 12905_2025_4253_MOESM3_ESM.docx]

**Additional File 3 – Results of subgroup analysis in NM**

Changes in Androgen Profile Over the Menstrual Cycle and Hormonal Contraceptive Phases in Physically Active Females

Vera M. Salmi^1^*, Ritva S. Mikkonen^1^, Ida E. Löfberg^1^, Kelly L. McNulty^2^, Kirsty M. Hicks^2,3^, Anthony C. Hackney^4^, Johanna K. Ihalainen^1,5^

1. Faculty of Sport and Health Sciences, University of Jyväskylä, Jyväskylä, Finland
2. Department of Sport, Exercise and Rehabilitation, Faculty of Health and Life Sciences, Northumbria University, Newcastle-upon-Tyne, UK
3. Performance, Medical and Innovation Department, Washington Spirit Soccer Club, Washington DC, USA
4. Department of Exercise & Sport Science – Department of Nutrition, University of North Carolina, Chapel Hill, North Carolina, USA
5. Finnish Institute of High Performance Sport KIHU, Jyväskylä, Finland

In NM participants with P4 > 16 nmol·L^−1^, concentrations of LH were significantly higher at ovulatory phase (M3) compared to bleeding (M1) (Supplementary Table S4), mid-follicular phase (M2) and mid-luteal phase (M4) (*p* < 0.001) and at M2 compared to M4 (*p* = 0.040) (Supplementary Fig. S2). In the analyses of androgens, concentrations of tT were significantly lower at M2 compared to M3 (*p* < 0.005) and M4 (*p* = 0.030) in participants who had serum concentrations of P4 > 16 nmol·L^−1^. In participants with concentrations of P4 > 16 nmol·L^−1^, significantly higher concentrations of fT were observed at M3 compared to M1 and to M4 (*p* < 0.001). Participants who had serum concentrations of P4 > 16 nmol·L^−1^ had also significantly higher concentrations of DHEA at M2 and at M3 compared to M1 and higher concentrations of DHEA-S at M2 compared to M1 (Supplementary Table S4).

In participants with concentrations of P4 ≤ 16 nmol·L^−1^, concentrations of LH were significantly higher only at M3 compared to M2 (*p* = 0.048) (Supplementary Fig. S2). Participants who had serum concentrations of P4 ≤ 16 nmol·L^−1^ had significantly higher concentrations of tT only at M3 compared to M2 (*p* = 0.006) and higher concentrations of fT at M3 compared to M1 (Supplementary Table S4). Concentrations of DHEA were lower at M2 compared to M4 (*p* = 0.027), while concentrations of DHEA-S remained unchanged throughout the MC in those with concentrations of P4 ≤ 16 nmol·L^−1^. Concentrations of SHBG remained unchanged throughout the MC in both subgroups. Median, 95% CI, individual hormone profiles of measured sex hormones and concentrations of SHBG, and p-values of post hoc analyses are presented in Supplementary Fig. S2.

**Supplementary Table S4 Changes in hormone and SHBG levels based on concentrations of P4 at M4 in NM**

|  | **M2 vs. M1** | | |  | **M3 vs. M1** | | |  | **M4 vs. M1** | | |
| --- | --- | --- | --- | --- | --- | --- | --- | --- | --- | --- | --- |
|  | **β (SE)** | **95% CI** | **P** |  | **β (SE)** | **95% CI** | **P** |  | **β (SE)** | **95% CI** | **P** |
| **P4 ≤ 16 nmol·l**^−^**^1^** |  |  |  |  |  |  |  |  |  |  |  |
| **E2 (pmol·L**^−^**^1^)** | 78.49 (104.02) | −125.40, 282.36 | 0.451 |  | 124.82 (167.00) | −202.50, 452.13 | 0.455 |  | 344.55 (90.62) | 166.95, 522.16 | **< 0.001** |
| **P4 (nmol·L**^−^**^1^)** | 0.10 (0.25) | −0.38, 0.58 | 0.685 |  | 3.16 (1.02) | 1.16, 5.15 | **0.002** |  | 4.52 (1.10) | 2.37, 6.68 | **< 0.001** |
| **LH (IU·L**^−^**^1^)** | 0.19 (1.36) | −2.47, 2.85 | 0.888 |  | 17.49 (9.11) | −0.37, 35.34 | 0.055 |  | 1.79 (1.78) | −1.70, 5.29 | 0.315 |
| **FSH (IU·L**^−^**^1^)** | 1.13 (0.81) | −0.46, 2.72 | 0.164 |  | 3.61 (1.85) | −0.01, 7.23 | 0.051 |  | −0.70 (0.72) | −2.11, 0.70 | 0.326 |
| **tT (nmol·L**^−^**^1^)** | −0.10 (0.12) | −0.33, 0.13 | 0.394 |  | 0.15 (0.13) | −0.11, 0.41 | 0.256 |  | 0.12 (0.13) | −0.14, 0.37 | 0.374 |
| **fT (pmol·L**^−^**^1^)** | −0.30 (1.02) | −2.30, 1.71 | 0.772 |  | 1.44 (0.55) | 0.37, 2.51 | **0.008** |  | 0.78 (0.55) | −0.29, 1.86 | 0.154 |
| **DHEA (nmol·L**^−^**^1^)** | −3.45 (3.63) | −10.57, 3.67 | 0.343 |  | 3.13 (5.02) | −6.72, 12.97 | 0.534 |  | 3.43 (4.15) | −4.70, 11.55 | 0.408 |
| **DHEA-S (µmol·L**^−^**^1^)** | 0.23 (0.17) | −0.11, 0.57 | 0.181 |  | 0.06 (0.42) | −0.78, 0.89 | 0.893 |  | 0.35 (0.29) | −0.21, 0.92 | 0.221 |
| **SHBG (nmol·L**^−^**^1^)** | −0.75 (2.66) | −5.96, 4.46 | 0.778 |  | 0.15 (2.03) | −3.83, 4.12 | 0.943 |  | 1.61 (2.27) | −2.83, 6.06 | 0.477 |
| **P4 > 16 nmol·l**^−^**^1^** |  |  |  |  |  |  |  |  |  |  |  |
| **E2 (pmol·L**^−^**^1^)** | 172.68 (28.60) | 116.63, 228.74 | **< 0.001** |  | 446.60 (85.41) | 279.21, 614.00 | **< 0.001** |  | 422.64 (40.58) | 343.11, 502.17 | **< 0.001** |
| **P4 (nmol·L**^−^**^1^)** | −0.45 (0.32) | −1.07, 0.17 | 0.157 |  | 2.40 (0.63) | 1.15, 3.64 | **< 0.001** |  | 22.69 (1.29) | 20.17, 25.21 | **< 0.001** |
| **LH (IU·L**^−^**^1^)** | 1.21 (0.62) | −0.02, 2.43 | 0.053 |  | 8.33 (1.41) | 5.57, 11.09 | **< 0.001** |  | −0.44 (0.97) | −2.34, 1.47 | 0.652 |
| **FSH (IU·L**^−^**^1^)** | 0.35 (0.66) | −0.94, 1.63 | 0.595 |  | 0.25 (0.54) | −0.80, 1.30 | 0.637 |  | −3.39 (0.69) | −4.74, −2.03 | **< 0.001** |
| **tT (nmol·L**^−^**^1^)** | −0.04 (0.09) | −0.23, 0.14 | 0.641 |  | 0.18 (0.10) | −0.03, 0.38 | 0.093 |  | 0.13 (0.10) | −0.07, 0.33 | 0.191 |
| **fT (pmol·L**^−^**^1^)** | 1.04 (0.58) | −0.10, 2.17 | 0.073 |  | 2.09 (0.57) | 0.97, 3.21 | **< 0.001** |  | −0.25 (0.66) | −1.54, 1.04 | 0.706 |
| **DHEA (nmol·L**^−^**^1^)** | 6.47 (2.67) | 1.24, 11.70 | **0.015** |  | 7.30 (3.11) | 1.21, 13.40 | **0.019** |  | 3.54 (3.09) | −2.52, 9.59 | 0.253 |
| **DHEA-S (µmol·L**^−^**^1^)** | 0.44 (0.18) | 0.09, 0.80 | **0.015** |  | 0.31 (0.19) | −0.07, 0.68 | 0.109 |  | 0.19 (0.20) | −0.21, 0.59 | 0.355 |
| **SHBG (nmol·L**^−^**^1^)** | 0.48 (1.39) | −2.25, 3.21 | 0.729 |  | 3.07 (2.03) | −0.91, 7.05 | 0.131 |  | 2.22 (1.92) | −1.54, 5.98 | 0.248 |

Values are presented as regression coefficients (β), standard errors (SE) and 95% confidence intervals (CI). E2, estradiol; P4, progesterone; LH, luteinizing hormone; FSH, follicle-stimulating hormone; tT, total testosterone; fT, free testosterone; DHEA, dehydroepiandrosterone; DHEA-S, dehydroepiandrosterone sulfate; SHBG, sex hormone-binding globulin; P4, progesterone; NM, naturally menstruating females; M1, bleeding; M2, mid-follicular phase; M3, ovulatory phase; M4, mid-luteal phase. Significant findings are denoted in bold

**Supplementary Figure S2**
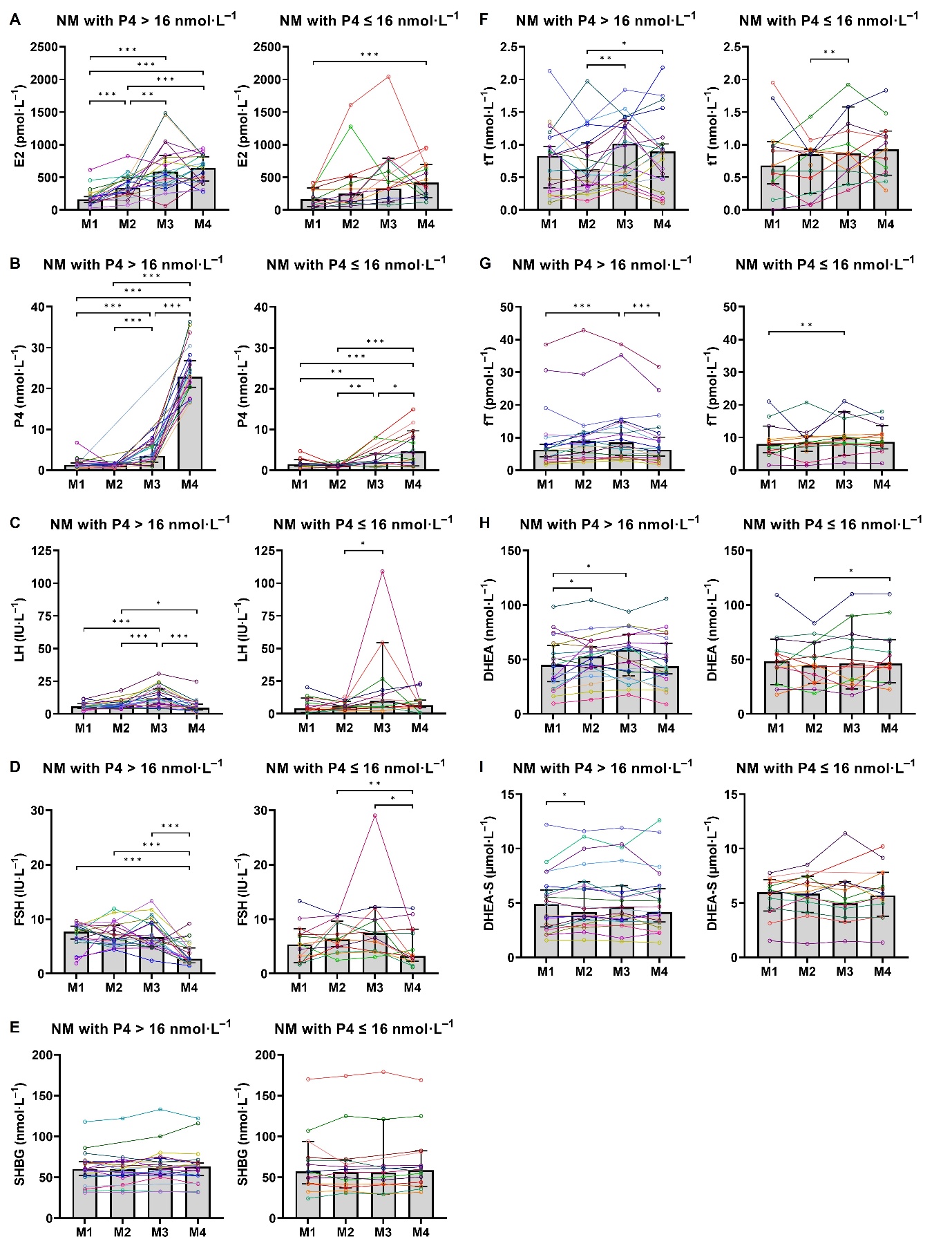


Changes in concentrations of hormones and SHBG based on P4 concentrations at M4 in NM. Median, 95% confidence interval, p-values of post hoc analyses and individual hormone profiles of **A)** estradiol (E2), **B)** progesterone (P4), **C)** luteinizing hormone (LH), **D)** follicle-stimulating hormone (FSH), **E)** sex hormone-binding globulin (SHBG), **F)** total testosterone (tT), **G)** free testosterone (fT), **H)** dehydroepiandrosterone (DHEA), and **I)** dehydroepiandrosterone sulfate (DHEA-S) for naturally menstruating females (NM; M1 = bleeding, M2 = mid-follicular phase, M3 = ovulatory phase, M4 = mid-luteal phase). Significant difference * = *p* ≤ 0.05, ** = *p* < 0.01, *** = *p* < 0.001
