# Supplementary material for: Changes in androgen profile over the menstrual cycle and hormonal contraceptive phases in physically active females
Source: BMC Womens Health. 2026 Jan 27;26:118. doi: 10.1186/s12905-025-04253-6 (PMC12918223; doi:10.1186/s12905-025-04253-6)
Supplement: Supplementary file 4 — Additional file 4 – Concentrations of E2, P4, LH and FSH between groups. [file 12905_2025_4253_MOESM4_ESM.docx]

**Additional File 4 – Concentrations of E2, P4, LH and FSH between groups**

Changes in Androgen Profile Over the Menstrual Cycle and Hormonal Contraceptive Phases in Physically Active Females

Vera M. Salmi^1^*, Ritva S. Mikkonen^1^, Ida E. Löfberg^1^, Kelly L. McNulty^2^, Kirsty M. Hicks^2,3^, Anthony C. Hackney^4^, Johanna K. Ihalainen^1,5^

1. Faculty of Sport and Health Sciences, University of Jyväskylä, Jyväskylä, Finland
2. Department of Sport, Exercise and Rehabilitation, Faculty of Health and Life Sciences, Northumbria University, Newcastle-upon-Tyne, UK
3. Performance, Medical and Innovation Department, Washington Spirit Soccer Club, Washington DC, USA
4. Department of Exercise & Sport Science – Department of Nutrition, University of North Carolina, Chapel Hill, North Carolina, USA
5. Finnish Institute of High Performance Sport KIHU, Jyväskylä, Finland

Naturally menstruating females (NM) had significantly higher concentrations of estradiol (E2) at M1 and M4 compared to corresponding measurements in hormonal intrauterine device using females (IUD) (β = 75.55, *p* = 0.012; β = 172.47, *p* = 0.019, respectively) and at M2, M3 and M4 compared to corresponding measurements in combined hormonal contraceptive using females (CHC) (β = 197.61, *p* = 0.003; β = 448.03, *p* < 0.001; β = 375.27, *p* < 001, respectively). CHC had significantly lower concentrations of E2 at M3 and M4 compared to IUD (β = −455.31, *p* = 0.002; β = −202.81, *p* = 0.002). Concentrations of progesterone (P4) were significantly lower in CHC at M1, M3 and M4 compared to NM (β = −0.62, *p* = 0.008; β = −2.54, *p* < 0.001; β = −15.63, *p* < 0.001) and at M4 compared to IUD (β = −14.42, *p* < 0.001). Luteinizing hormone (LH) was significantly lower in CHC in all measurement points compared to NM (β = −2.35, *p* = 0.017; β = −3.60, *p* < 0.001; β = −15.54, *p* < 0.001, β = −3.85; *p* = 0.002) and at M2, M3 and M4 compared to IUD (β = −6.90, *p* = 0.010; β = −11.44, *p* < 0.001; β = −3.67, *p* = 0.015). Follicle-stimulating hormone (FSH) was significantly lower in CHC at M1, M2 and M3 compared to NM (β = −2.41, *p* = 0.001; β = −4.73, *p* < 0.001; β = −6.10, *p* < 0.001) and IUD (β = −1.74, *p* = 0.021; β = −5.39, *p* < 0.001; β = −5.40, *p* < 0.001).
