# Supplementary material for: Changes in androgen profile over the menstrual cycle and hormonal contraceptive phases in physically active females
Source: BMC Womens Health. 2026 Jan 27;26:118. doi: 10.1186/s12905-025-04253-6 (PMC12918223; doi:10.1186/s12905-025-04253-6)
Supplement: Supplementary file 5 — Additional file 5 – Changes in E2, P4, LH and FSH in subgroup analysis of IUD. Supplementary Table S5 Changes in E2, P4, LH and FSH levels in participants using Jaydess or Kyleena and Mirena intrauterine device. [file 12905_2025_4253_MOESM5_ESM.docx]

**Additional File 5 – Changes in E2, P4, LH and FSH in subgroup analysis of IUD**

Changes in Androgen Profile Over the Menstrual Cycle and Hormonal Contraceptive Phases in Physically Active Females

Vera M. Salmi^1^*, Ritva S. Mikkonen^1^, Ida E. Löfberg^1^, Kelly L. McNulty^2^, Kirsty M. Hicks^2,3^, Anthony C. Hackney^4^, Johanna K. Ihalainen^1,5^

1. Faculty of Sport and Health Sciences, University of Jyväskylä, Jyväskylä, Finland
2. Department of Sport, Exercise and Rehabilitation, Faculty of Health and Life Sciences, Northumbria University, Newcastle-upon-Tyne, UK
3. Performance, Medical and Innovation Department, Washington Spirit Soccer Club, Washington DC, USA
4. Department of Exercise & Sport Science – Department of Nutrition, University of North Carolina, Chapel Hill, North Carolina, USA
5. Finnish Institute of High Performance Sport KIHU, Jyväskylä, Finland

**Supplementary Table S5 Changes in E2, P4, LH and FSH levels in participants using Jaydess or Kyleena and Mirena intrauterine device**

|  | **Jaydess & Kyleena (n = 6)** | | |  | **Mirena (n = 6)** | | |
| --- | --- | --- | --- | --- | --- | --- | --- |
|  | **M2 vs. M1** | **M3 vs. M1** | **M4 vs. M1** |  | **M2 vs. M1** | **M3 vs. M1** | **M4 vs. M1** |
| **E2 (nmol·L^−1^)** |  |  |  |  |  |  |  |
| **β (SE)** | 85.55 (51.73) | **611.38 (212.41)** | **331.22 (60.56)** |  | 179.25 (121.14) | **401.97 (151.27)** | **197.30 (58.05)** |
| **95% CI** | −15.84, 186.94 | **195.06, 1027.71** | **212.53, 449.91** |  | −58.18, 416.68 | **105.48, 698.45** | **83.52, 311.08** |
| **P** | 0.098 | **0.004** | **< 0.001** |  | 0.139 | **0.008** | **< 0.001** |
| **P4 (pmol·L^−1^)** |  |  |  |  |  |  |  |
| **β (SE)** | **−1.05 (0.48)** | 0.72 (0.93) | **21.43 (5.06)** |  | −0.25 (0.74) | 5.42 (5.01) | **6.21 (2.81)** |
| **95% CI** | **−2.00, −0.11** | −1.09, 2.54 | **11.52, 31.34** |  | −1.70, 1.20 | −4.40, 15.24 | **0.70, 11.72** |
| **P** | **0.029** | 0.435 | **< 0.001** |  | 0.736 | 0.279 | **0.027** |
| **LH (IU·L^−1^)** |  |  |  |  |  |  |  |
| **β (SE)** | **1.37 (0.68)** | **12.49 (3.09)** | 2.67 (1.62) |  | **8.58 (3.86)** | 4.09 (2.76) | 0.08 (1.03) |
| **95% CI** | **0.03, 2.70** | **6.44, 18.55** | −0.50, 5.84 |  | **1.03, 16.14** | −1.32, 9.51 | −1.93, 2.10 |
| **P** | **0.045** | **< 0.001** | 0.099 |  | **0.026** | 0.138 | 0.934 |
| **FSH (IU·L^−1^)** |  |  |  |  |  |  |  |
| **β (SE)** | **1.26 (0.59)** | **2.45 (0.91)** | **−1.36 (0.69)** |  | **2.44 (0.76)** | 0.54 (1.07) | −0.747 (1.58) |
| **95% CI** | **0.11, 2.42** | **0.68, 4.23** | **−2.72, −0.002** |  | **0.96, 3.93** | −1.57, 2.64 | −3.84, 2.35 |
| **P** | **0.032** | **0.007** | **0.050** |  | **0.001** | 0.617 | 0.637 |

Values are presented as regression coefficients (β), standard errors (SE) and 95% confidence intervals (CI). E2, estradiol; P4, progesterone; LH, luteinizing hormone; FSH, follicle-stimulating hormone; M1, bleeding or lowest E2 concentration and/or sample after highest P4 concentration; M2, M1 + 7 days; M3, M1 + 14 days; M4, M1 + 21 days. Significant findings are denoted in bold.
